# The Influence of Different Fat Sources on Steatohepatitis and Fibrosis Development in the Western Diet Mouse Model of Non-alcoholic Steatohepatitis (NASH)

**DOI:** 10.3389/fphys.2019.00770

**Published:** 2019-06-25

**Authors:** Hannah K. Drescher, Ralf Weiskirchen, Annabelle Fülöp, Carsten Hopf, Estibaliz González de San Román, Pitter F. Huesgen, Alain de Bruin, Laura Bongiovanni, Annette Christ, René Tolba, Christian Trautwein, Daniela C. Kroy

**Affiliations:** ^1^Department of Internal Medicine III, University Hospital RWTH Aachen, Aachen, Germany; ^2^Institute of Molecular Pathobiochemistry, Experimental Gene Therapy and Clinical Chemistry (IFMPEGKC), University Hospital RWTH Aachen, Aachen, Germany; ^3^Center for Biomedical Mass Spectrometry and Optical Spectroscopy (CeMOS), Mannheim University of Applied Sciences, Mannheim, Germany; ^4^Central Institute for Engineering, Electronics and Analytics, ZEA-3 – Forschungszentrum Jülich, Jülich, Germany; ^5^Department of Pathobiology, Faculty of Veterinary Medicine, Utrecht University, Utrecht, Netherlands; ^6^Institute of Innate Immunity, University Hospital Bonn, Bonn, Germany; ^7^Department of Infectious Diseases and Immunology, University of Massachusetts Medical School, Worcester, MA, United States; ^8^Institute of Laboratory Animal Science and Experimental Surgery and Central Laboratory for Laboratory Animal Science, University Hospital RWTH Aachen, Aachen, Germany

**Keywords:** non-alcoholic steatohepatitis, Western diet, fatty liver, fibrosis, animal model and liver injury

## Abstract

Non-alcoholic steatohepatitis (NASH) is the leading cause of chronic liver injury and the third most common reason for liver transplantations in Western countries. It is unclear so far how different fat sources in Western diets (WD) influence the development of NASH. Our study investigates the impact of non-trans fat (NTF) and corn oil (Corn) as fat source in a WD mouse model of steatohepatitis on disease development and progression. C57BL/6J wildtype (WT) mice were fed “standard” WD (WD-Std), WD-NTF or WD-Corn for 24 weeks. WT animals treated with WD-NTF exhibit distinct features of the metabolic syndrome compared to WD-Std and WD-Corn. This becomes evident by a worsened insulin resistance and elevated serum ALT, cholesterol and triglyceride (TG) levels compared to WD-Corn. Animals fed WD-Corn on the contrary tend to a weakened disease progression in the described parameters. After 24 weeks feeding with WD-NTF and WD-Std, WD-Corn lead to a comparable steatohepatitis initiation by histomorphological changes and immune cell infiltration compared to WD-Std. Immune cell infiltration results in a significant increase in mRNA expression of the pro-inflammatory cytokines IL-6 and TNF-α, which is more pronounced in WD-NTF compared to WD-Std and WD-Corn. Interestingly the fat source has no impact on the composition of accumulating fat within liver tissue as determined by matrix-assisted laser desorption/ionization mass spectrometry imaging of multiple lipid classes. The described effects of different fat sources on the development of steatohepatitis finally resulted in variations in fibrosis development. Animals treated with WD-NTF displayed massive collagen accumulation, whereas WD-Corn even seems to protect from extracellular matrix deposition. Noteworthy, WD-Corn provokes massive histomorphological modifications in epididymal white adipose tissue (eWAT) and severe accumulation of extracellular matrix which are not apparent in WD-Std and WD-NTF treatment. Different fat sources in WD-Std contribute to strong steatohepatitis development in WT mice after 24 weeks treatment. Surprisingly, corn oil provokes histomorphological changes in eWAT tissue. Accordingly, both WD-NTF and WD-Corn appear suitable as alternative dietary treatment to replace “standard” WD-Std as a diet mouse model of steatohepatitis whereas WD-Corn leads to strong changes in eWAT morphology.

## Introduction

Non-alcoholic fatty liver disease (NAFLD) is one of the most prevalent chronic liver diseases. It is the third most common reason for liver transplantations in industrialized countries and is in progress to get the second leading etiology of liver disease (Wong et al., 2015). NAFLD is strongly associated with mortality and encompasses a sequence of pathological changes within the liver (Yamaguchi et al., 2007; Estes et al., 2018a; Younossi et al., 2018a,b). These hepatic alterations in general begin with simple steatosis and evolve to more advanced stages like Non-alcoholic steatohepatitis (NASH), cirrhosis and end stage diseases such as hepatocellular carcinoma (HCC) (Browning and Horton, 2004; Clark, 2006). Studies indicate that nearly 20% of the general population in western countries suffer from fatty liver disease and bear the risk to develop NASH (Wong et al., 2014). Recent calculations even predict that the incidence of HCC and NASH-related liver transplantations will increase more than 100% till 2030 (Estes et al., 2018b). As hepatic manifestation of the metabolic syndrome NASH frequently occurs together with obesity due to poor lifestyle and wrong dietary fat consumption, type II diabetes, dyslipidemia but also genetic and epigenetic factors (Rice Bradley, 2018; Schuppan et al., 2018). It is further accompanied with insulin resistance.

On the molecular level NASH is associated with an impaired lipid metabolism. This leads to an enhanced release of free fatty acids (FFA) from skeletal muscle and visceral adipose tissue leading to fat droplet accumulation in hepatocytes (Choi and Diehl, 2008). These FFA in turn disturb *de novo* lipogenesis and alter β-oxidation (Capurso and Capurso, 2012). A low amount of dietary polyunsaturated fatty acids (PUFA), especially n-3 PUFA but not n-6 PUFA, was shown to be associated with the development of NAFLD including typical features of the metabolic syndrome by regulating the expression of transcription factors involved in fatty acids oxidation (Pachikian et al., 2011). Conversely, the supplementation of n-3 PUFA leads to amelioration of hepatic steatosis (Levy et al., 2004; Capanni et al., 2006).

Further characteristics of NAFLD activity within liver tissue are the ballooning of hepatocytes and inflammatory cell invasion (Kleiner et al., 2005; Hubscher, 2006). The development from simple steatosis to steatohepatitis is further linked to oxidative stress induced by toxic lipids and high dietary cholesterol with an increase in inflammatory cytokine production finally leading to the progression to fibrosis (Wouters et al., 2008, 2010; Jump et al., 2016). At the given rise of obese patients worldwide also the prevalence of patients developing fatty liver diseases is increasing dramatically thus getting a global socio-economic health burden (Agopian et al., 2012).

Up to the current moment there exists no targeted therapy expect of changes in lifestyle to cure and prevent from NAFLD (Neuschwander-Tetri, 2009). Since 2003 partially hydrogenated oils or trans fats have been reported to be directly linked to cardiovascular disease for a daily intake as little as 5 g (Mozaffarian et al., 2006). These publications and a court process against Kraft concerning trans fats in Oreo cookies opened the debate how to officially handle the use of trans fats in food industry^1^. Based on this literature the Food and Drug Administration (FDA) reacted with a labeling plan by which a trans fat content ≥ 0.5 g has to be listed on food nutrition labels in the same year. Already in 2003 Denmark was the first nation worldwide widely banning trans fats leading the World Health Organization (WHO) to establish a recommendation for a daily limit of trans fat intake in 2004. Several big international fast-food chains increasingly use trans fat free oil in their restaurants on a voluntary basis.

Based in these developments we critically reconsidered the composition of the general used WD containing trans fat shortening as fat source leading to the development of the metabolic syndrome and steatosis related liver damage. Our aim was to investigate the ability and the impact of different fat sources in WD regarding their potential to initiate diet-induced steatohepatitis in male C57/B6J mice. In two distinct experiments we tried to induce murine steatohepatitis by feeding a trans fat-free WD (WD-NTF) and a WD containing corn oil as fat source (WD-Corn). Surprisingly a high amount of corn oil in WD is associated with severe histomorphological changes in eWAT tissue and thereby exhibits the risk to cause critical metabolic changes. Interestingly, this study further shows that the type of fat source does not have significant influence on the composition of fat accumulating in the liver during progression and manifestation of steatohepatitis.

## Materials and Methods

### Ethics Statement

This study was performed in strict accordance with the recommendations of the Ethics of the regional authorities for nature, environmental and consumer protection of North Rhine-Westfalia (LANUV, Landesamt für Natur, Umwelt und Verbraucherschutz NRW) Recklinghausen, Germany, and approved by the LANUV Committee (Permit Number: TV11018G). Further, all experiments were performed in accordance with the German guidelines for animal housing and husbandry.

### Housing and Generation of Mice

Male wildtype mice (C57BL/6J) were purchased from Janvier Labs, Le Genest-Saint-Isle, France. According to the EU-Directive 2010/63 (Liedtke et al., 2013), we have tried to use minimum numbers of animals for our animal experiments. This Directive demands that all experiments with animals must be performed in an ethical framework, which is based primarily on the replacement, refinement and reduction principle (i.e., the 3R principle) and first proposed 60 years before (Russell and Burch, 1959). In our experiments, 4 mice per group were fed and analyzed in parallel experiments. Animals were housed in the animal facility of the University Hospital RWTH Aachen in 12-h light/dark cycles with water and food *ad libitum* available. All animals were treated in accordance with the criteria of the German administrative panel on laboratory animal care.

### WD Treatment

For dietary treatment 8–12 weeks old male C57BL/6J mice were either fed standard Western diet (WD-Std) (40 kcal % fat (Primex Shortening), 20 kcal % fructose, 2% cholesterol) (cat. no. D09100301), WD with non-trans fat Primex Shortening as fat source (WD-NTF) (40 kcal % fat (Non trans fat Primex Shortening), 20 kcal % fructose, 2% cholesterol) (cat. no. D16022301), WD with corn oil as fat source (WD-Corn) (40 kcal % fat (corn oil), 20 kcal % fructose, 2% cholesterol) (cat. no. 16010101). The different diets are marked by the supplier in different color codes (brown, white, red, and blue). All animals were critically inspected each day to exclude differences in food intake caused by triturating without consuming. All animals showed a daily food intake of 5–6 g without differences between chow and dietary treatment. To alleviate unnecessary suffering, animals were sacrificed at the end of dietary treatment by cervical dislocation.

### Quantification and Statistics

All results were expressed as mean ± SEM and represent data from 4 animals per group and time point. Calculations via manual counting of positive cells were done in 10 high power fields per liver. *p*-values were measured using Student’s *t*-test when comparing two groups or ANOVA testing with Tukey’s multiple comparison post-test. A value of *p* < 0.05 was considered significant (^∗^*p* < 0.05, ^∗∗^*p* < 0.01, ^∗∗∗^*p* < 0.001).

### Blood Collection and Serum Analytics

Blood collection was performed retro-orbital. Therefore, mice were shortly anesthetized with isoflurane and blood was collected via a glass capillary. Samples were centrifuged at 10,000 rpm for 5 min and serum was stored at –80°C. Serum transaminase levels [aspartate aminotransferase (AST) and serum alanine aminotransferase (ALT)] were processed by the Central Laboratory Facility (LDZ) of the University Hospital RWTH Aachen.

### NAFLD Activity Score

Histopathological scoring was done by Prof. Dr. Alain de Bruin at the University of Utrecht via a NAFLD activity score (NAS) as described by Kleiner et al. (2005) and Hubscher (2006).

### Glucose Tolerance Test

Animals were fasted for 6 h and blood glucose was measured every 30 min following intraperitoneal administration of 0.2 g/kg glucose for 2 h.

### Hepatic Triglycerides

Hepatic triglyceride levels were analyzed by weighing 20 mg snap frozen liver tissue. This tissue was homogenized in 1 ml of buffer (10 mM Tris, 2 mM EDTA, 0.25 M sucrose, pH 7.5). A standard curve was calculated in accordance with the manufacturer’s instructions of the Instruchemie liquicolor mono kit (InstruChemie, Delfzijl, Netherlands). 200 μl of the kit reagent were added to 2 μg of the sample or the standard solution and incubated for 45 min at room temperature. After that the OD was measured at 492 nm.

### Histology, Sirius-Red Staining, Oil Red O Staining

Liver samples were fixed in 4% formaldehyde, embedded in paraffin, cut and stained with hematoxylin and eosin. Pictures were taken using an Axio-Imager Z1 (Carl Zeiss, Jena, Germany) for each treatment per genotype. For Sirius-red staining the paraffin sections were incubated in a solution containing 0.1% Sirius red and 0.1% picric acid (pH 2.0) for 1 h. After that slides were incubated in 0.1 M HCl for 5 min, treated with an ascending ethanol series and finally incubated and covered in Roti-Histol (Roth, Karlsruhe, Germany). 10 images per liver section were analyzed under polarized light. Photomicrographs of Sirius-red positive areas taken in a 400× magnification were analyzed via color error measurement using the open source software Image-J. For Oil Red O staining formalin fixed frozen liver sections were washed in PBS and stained with Oil Red O staining solution (Sigma-Aldrich, Taufkirchen, Germany, cat. no. O1391). After rinsing with water counterstaining of nuclei was performed with hematoxylin.

### Immunofluorescent Stainings

Liver tissue samples conserved in OCT compound were cut in 5-mm sections, air-dried and fixed with ice-cold 4% paraformaldehyde. Antibodies (Supplementary Table S1) were incubated in 1% mouse serum dissolved in PBS (PAA, Vienna, Austria) containing 0.02% sodium acetate (Sigma-Aldrich) for 1 h at room temperature. AlexaFluor 488-conjugated or Alexa Fluor 594-conjugated secondary antibodies (Molecular Probes, Boston, MA, United States) were used for detection. Nuclei were counterstained with DAPI (Vector Laboratories/Axxora, Loerrach, Germany). Immunofluorescent signals were detected using an AxioImager Z1 microscope (Carl Zeiss, Jena, Germany). Images were taken in a 400× magnification. 10 photographs per mouse liver with at least 4 mice per group were counted.

### Gene Expression Analysis by Real-Time PCR

Total mRNA was extracted from whole cryopreserved liver tissue by using the peqGOLD RNAPure^TM^ kit (Peqlab, Erlangen, Germany) according to the manufacturer’s recommendations. For cDNA synthesis 500–1,000 ng of total mRNA was first digested with DNAse I with the DNAse I kit (Invitrogen, Karlsruhe, Germany) and subsequently reverse-transcribed with the Omniscript reverse-transcription kit (Qiagen, Hilden, Germany) according to the manufacturer’s instructions and used in Real-Time PCR (Applied Biosystems, Foster City, CA). The detection gene expression was performed by using the SybrGreen tqPCR Supermix (Invitrogen). Primer sequences can be found in Supplementary Table S2. The mRNA extracts from 4 mice per group were analyzed individually. The PCR efficiencies in our real-time PCR were evaluated by the 2^-ΔΔCt^ method (Livak and Schmittgen, 2001).

### Mouse Serum MultiPlex Cytokine Measurements

Cytokines were measured in mouse sera using the chemokine 9-Plex mouse PorcartaPlex^TM^ bead assay (Thermo Fisher Scientific, Schwerte, Germany) and a MAGPIX instrument using Luminex xMAP Technology. The analysis was essentially performed according to the manufacturer’s protocol.

### Isolation of Cells and Flow Cytometry

Leukocytes were isolated from whole liver extracts. To analyze leukocytes in whole liver extracts livers were perfused with 10 ml phosphate-buffered saline (PBS), cut up with scissors and digested with collagenase IV (Worthington Biochemical Corporation, Lakewood, NJ, United States) at 37°C for 30 min. Digested liver extracts were filtered through a 70 μm cell strainer and cells were stained for 20 min at 4°C with fluorochrome-labeled monoclonal antibodies (used panel are depicted in Supplementary Table S3). Then, cells were subjected to flow cytometry using a BD Fortessa (BD Biosciences, Heidelberg, Germany). Data were analyzed using FlowJo software (TreeStar, Ashland, OR, United States).

### SDS PAGE and Western Blot

Tissue of snap frozen livers was lysed in ice cold lysis buffer (Supplementary Table S4). The protein lysat was heat denaturated for 5 min. at 95°C in double-strength sodium dodecyl sulfate sample buffer containing dithiothreitol before resolution in 10% SDS-PAGE. For primary antibody incubation membranes were probed with anti-α-SMA (ab32575, Abcam, Cambridge, United Kingdom), and anti-GAPDH (MCA4739, AbD serotec, Hercules, CA, United States) antibodies. As secondary antibodies HRP-linked anti-rabbit immunoglobulin G (7074S, Cell signaling, Frankfurt, Germany) and HRP-linked anti-rat (559286, BD Pharmingen, Heidelberg, Germany) were used. The antigen-antibody complexes were visualized using the ECL chemiluminescence kit (GE Healthcare, Buckinghamshire, United Kingdom).

### Sample Preparation for MALDI-MSI

Thin tissue sections were thawed at room temperature for 2–3 h inside a sealed box with silica gel to avoid accumulation of condensing water, followed by 30 min drying in a vacuum desiccator. The analysis was performed similar to the procedure described by Jackson et al. (2005). In detail, 20 mg/ml DHB in 50% ethanol was directly applied as matrix substance and deposited in 24 layers using a spray device (Suncollect sprayer, SunChrom GmbH, Friedrichsdorf, Germany). The first layer was deposited at 10 μl/min, the second at 20 μl/min, the third at 25 μl/min, and the following layers at 30 μl/min, with a final amount of 140.6 ± 3.2 μg/cm^2^.

### MALDI-MSI Measurement

A MALDI LTQ-Orbitrap XL hybrid mass spectrometer (Thermo Fisher Scientific, Bremen, Germany) equipped with a nitrogen laser (337 nm, rep. rate = 60 Hz, spot size = 80 × 120 μm) was used for mass analysis. The instrument was externally calibrated using commercial peptide standard mixtures (ProteoMass calibration kit, Sigma-Aldrich) for either the normal (m/z 150–2,000) or high (m/z 200–4,000) mass range. Xcalibur (version 2.3) from Thermo Fisher Scientific was used for MALDI-MSI data acquisition in positive and negative ion mode. The ion mass range was set to 400–1,000 Da in positive ion mode and 750–2,000 in negative ion mode, with 10 laser shots per step at laser energy of 15 μJ. The target plate stepping distance was set to 100 μm for both the *x*- and *y*-axes. The mass resolution was 100,000 (full width at half maximum at m/z 400).

### MALDI Data Analysis

Lipid species were assigned by comparison of the measured molecular masses to the Lipid Metabolomics and Pathways Strategy (MAPS) database^2^ and the Madison Metabolomics Consortium Database^3^. For assignment, a maximum of 5 ppm deviation between measured and theoretical mass was selected as the tolerance window. Due to the presence of salts in biological tissues, mass spectra will contain adducts of cationic salts, such as sodium or potassium apart from the protonated molecular ion [M+H]. Images and statistical analysis were carried out with SCiLS software lab 2018b (SCiLS, Bremen, Germany). First, raw data were converted to a suitable format for SCiLS, and normalized using the total ion count (TIC). The region of interest (ROI) where selected in the Nissl staining. A receiver-operator characteristic (ROC) analysis was done on each ROI, to statistically analyze m/z values that were either increased or decreased in the livers with different diets. The ROC analysis results were analyzed with Kruskal–Wallis test to find m/z values that were consistently statistically significant, in each ROI, across all datasets. Distribution maps of these selected m/z values were generated using SCiLS, with edge-preserving weak image denoising.

## Results

### Different Fat Sources in Western Diet Show Adverse Effects on the Outcome of the Metabolic Syndrome in Mice

**FIGURE 1:**
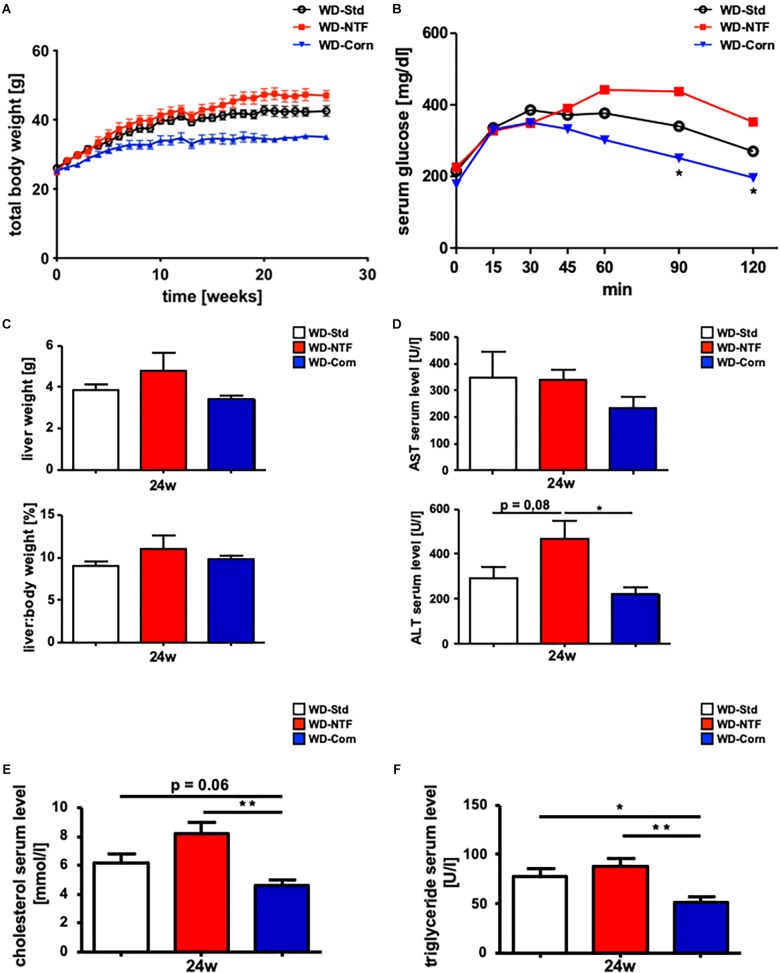
Non trans fat sources in WD cause impaired insulin resistance and increased serum parameters. **(A)** Total body weight curve during the 24 weeks feeding period with Western diets containing different fat sources (WD-Std, WD-NTF, WD-Corn) (*n* = 4). **(B)** After WD treatment for 24 weeks, animals getting a non-trans fat Western diet displayed significantly increased serum glucose levels during a glucose tolerance test for the assessment of insulin resistance (*n* = 4) (^∗^*p* < 0.05). **(C)** Total liver weight and liver:body weight ratio of WT animals after 24 weeks WD feeding (WD-Std, WD-NTF, WD-Corn) (*n* = 4). **(D)** AST and ALT levels in serum of WT animals after WD treatment for 24 weeks (WD-Std, WD-NTF, WD-Corn) (*n* = 4) (^∗^*p* < 0.05). **(E)** Serum cholesterol levels of WT animals after treatment with steatosis-inducing diets (WD-Std, WD-NTF, WD-Corn) (*n* = 4) (^∗∗^*p* < 0.01). **(F)** Serum TG levels of WT animals after 24 weeks feedings Western diets with different fat sources (WD-Std, WD-NTF, WD-Corn) (*n* = 4) (^∗^*p* < 0.05, ^∗∗^ *p* < 0.01).

The development of steatosis under WD-Std treatment is in part dependent on a high amount of fat within the diet. In a second experimental setup, we next aimed to analyze whether the source of fat within the WD influences the outcome of the metabolic syndrome and disease progression. Twelve weeks old WT mice (25 g) were fed standard WD (WD-Std), WD with a non-trans fat source (WD-NTF) and WD with corn oil as fat source (WD-Corn) for 24 weeks *ad libitum* that are marked by the supplier in a different color code (i.e., white, red, and blue) (Supplementary Figures S1–S3). Interestingly, mice fed with a WD containing corn oil as fat source trended to gain less weight over a 24 weeks treatment period (Figure 1A), while having the same food intake compared to the other groups (WD-Std, WD-NTF). The investigation of glucose tolerance revealed ameliorated glucose tolerance in the WD-Corn group, while WD-NTF treatment led to higher serum glucose levels over time (Figure 1B). In contrast, measurement of the total liver weight and a calculation of the liver:body weight ratio showed no differences between groups (WD-Std, WD-NTF, WD-Corn) (Figure 1C). Analysis of serum transaminases unexpectedly showed decreased AST and ALT levels in mice fed with WD-Corn compared to the other groups (Figure 1D). Further treatment with WD-Corn lead to dropped cholesterol and TG levels in serum compared to WD-Std and WD-NTF (Figure 1E,F).

### The Type of Fat Source Does Not Influence the Type of Fat Accumulating in Liver Tissue in WD-Induced Steatosis

**FIGURE 2:**
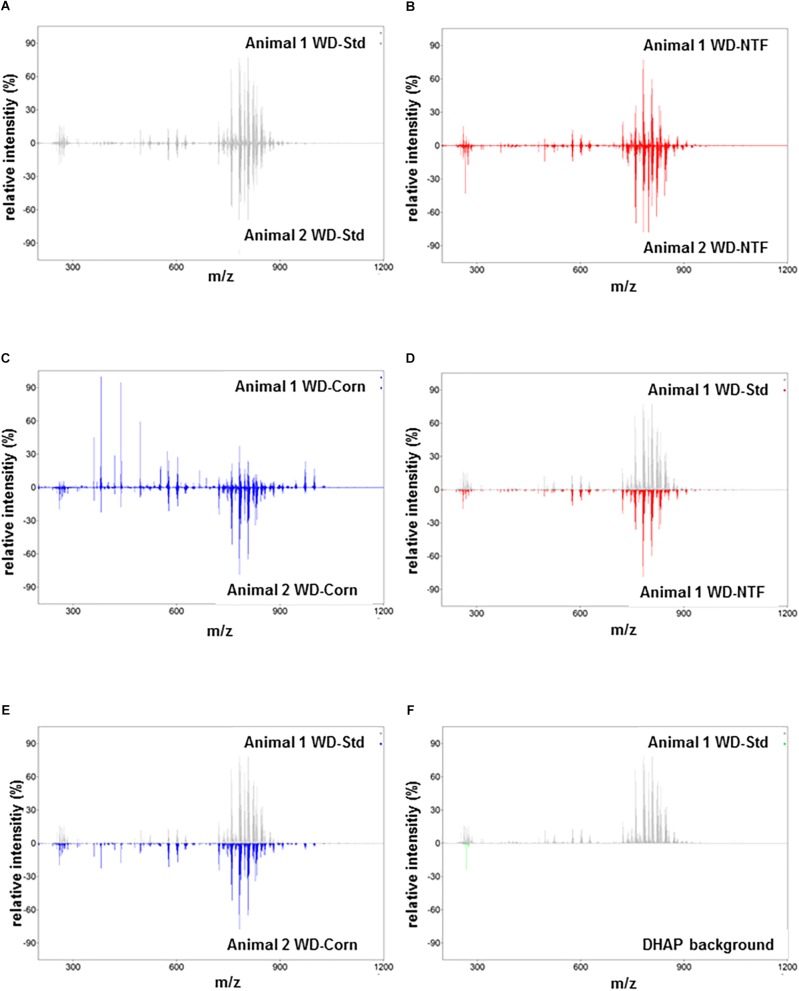
MALDI FT-ICR mass spectrometry imaging of multiple lipid classes. **(A–C)** Representative average spectra on liver tissue of animals that received **(A)** the WD-Std, **(B)** the WD-NTF, and **(C)** the WD-Corn. The low variation in relative peak intensities depicted above and below the zero line marks the low variability in the respective groups. **(D,E)** Comparative analysis of the relative m/z intensities between animals receiving **(D)** the WD-Std and WD-NTF, or animals that received **(E)** the WD-Std and WD-Corn, respectively. **(F)** To demonstrate the specificity of signals, the obtained m/z intensities of sections from an animal that received the WD-Std were compared to the 2,5-DHAP matrix background. Each average spectrum represents one animal measured by MALDI FT-ICR mass spectrometry imaging in positive ion mode.

**FIGURE 3:**
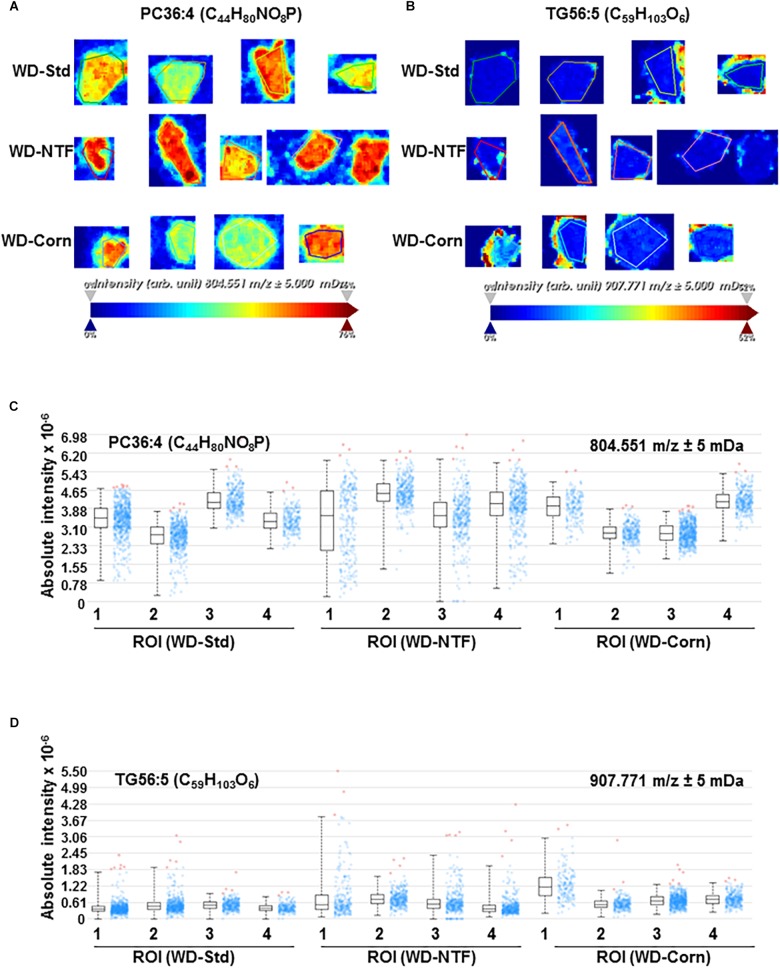
Comparison of individual lipid concentration and distribution by high resolution mass spectrometry imaging. Lipid composition in the liver of animals receiving the WD-Std, WD-NTF or WD-Corn compositions were assessed by mass spectrometry imaging (*n* = 4). Exemplary images of panel **(A)** phosphatidylcholine 36:4 (PC(36:4)) and **(B)** TG 56:5 (TG(56:5)) and corresponding quantified signal intensities of panel **(C)** PC(36:4) and **(D)** TG(56:5) are shown. Among all observed lipid species, no significantly different accumulation was found.

Next, we addressed the question whether WD treatment with different fat sources influences the composition of the fat accumulating in liver tissue during steatosis development. Therefore, we performed high-resolution matrix-associated laser desorption/ionization mass spectrometry imaging on liver specimens of mice fed with respective diets. Using this imaging technique, we were able to identify and classify the spatial distribution of thousands of lipids within the liver tissues. In regard to reproducibility, the molecules and their concentrations within the animals of the different groups were highly reproducible (Figure 2A–C). In addition, when comparing animals fed with different WD diets, the overall signals were the same (Figure 2D,E). The identified lipid signals during imaging were highly specific and not influenced by the 2,5-Dihydroxyacetophenone (DHAP) used as matrix (Figure 2F). Also, when we imaged individual classes of lipids in more detail, we found that the overall concentrations of defined lipids were the same in the different groups receiving different WDs (Figure 3 and Supplementary Figure S4).

### Different Fat Sources Lead to Altered Expression of Steatohepatitis and Fibrosis

**FIGURE 4:**
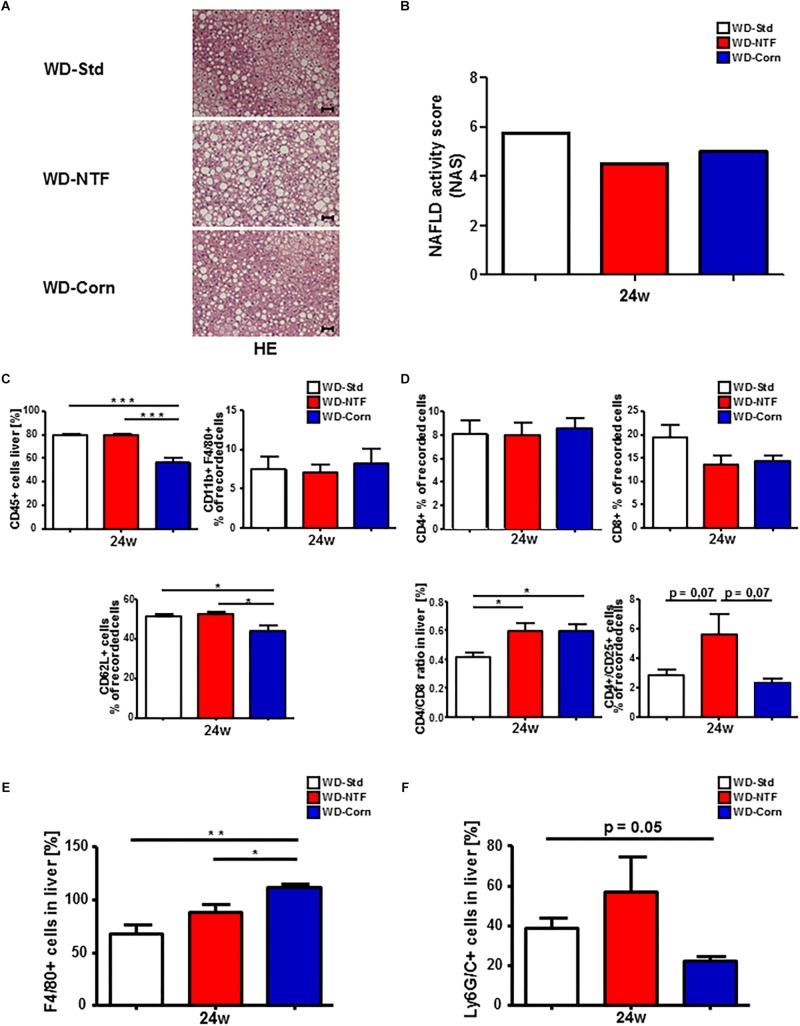
Differences in fat deposition and immune cell infiltration in Western diets with varying fat sources. **(A)** Representative H&E stained liver sections of WT animals after 24 weeks treatment with different Western style diets (WD-Std, WD-NTF, WD-Corn). **(B)** No differences in the NAFLD activity score (NAS) between WD with different fat sources. The used NAS considers steatosis, lobular inflammation and hepatocellular ballooning. Biopsies with a score < 3 were diagnosed “not NASH.” The histopathological validation was performed by Prof. Alain de Bruin at the University of Utrecht. **(C)** Flow cytometric analysis of CD11b^+^ cell subsets (CD11b^+^/F4/80^+^) after 24 weeks WD treatment. Cells were gated by FSC/SSC, duplets were excluded. Live/CD45^+^, CD11b^+^, CD11b^+^/F4/80^+^ (regarded as macrophages), CD62L^+^ (neutrophil infiltration and activation marker) (*n* = 4) (^∗^*p* < 0.05, ^∗∗∗^ *p* < 0.001). **(D)** Intrahepatic CD4^+^ and CD8^+^ T cells were analyzed by Flow Cytometry after feeding Western diets with different fat sources for 24 weeks. CD4^+^ and CD8^+^ T cells were gated by FSC/SSC, duplets were excluded, Live/CD45^+^, CD4^+^ or CD8^+^. A statistical analysis of the ratio of CD4^+^/CD8^+^ T cells of recorded cells was performed. Further CD4^+^ T cell subsets were gated via CD4^+^/CD25^+^ (regarded as regulatory T cells) (*n* = 4) (^∗^*p* < 0.05). **(E)** Statistical analysis of the percentage of F4/80^+^ cells on stained liver sections of WT mice after 24 weeks treatment with different WDs. 10 view fields/liver of *n* = 4 animals per genotype and time point were included (Scale bars: 100 μm, Magnification: 200×) (^∗^*p* < 0.05, ^∗∗^ *p* < 0.01). **(F)** Statistical analysis of the percentage of Ly6G^+^ cells on stained liver sections of WT mice after 24 weeks treatment with different WDs. 10 view fields/liver of *n* = 4 animals per genotype and time point were included (Scale bars: 100 μm, Magnification: 200×).

Twenty-four weeks WD treatment with different fat sources lead to strong liver injury shown by hepatocyte ballooning and massive fatty liver degeneration with fine and coarse fat droplet formation, getting evident in the hematoxylin and eosin (H&E) and Oil Red staining and in the direct measurement of intrahepatic TG (Figure 4A and Supplementary Figure S5). The histological scoring by the calculation of a NAFLD activity score (NAS) supports these findings (Figure 4B and Supplementary Figure S6). Invasive fat accumulation further provoked strong immune cell infiltration and subsequent inflammation in WD fed mice. Unexpectedly, the overall number of CD45^+^ cells was significantly decreased in mice fed with WD containing corn oil (Figure 4C). Analysis of myeloid derived immune cell subpopulations interestingly showed no significant differences in the amount of CD11b^+^/F480^+^ cells between treatment groups (for representative gating see Supplementary Figure S7A). The observed decrease was then reflected in CD62L^+^ cell populations. Next, we assessed hepatic infiltration of lymphoid immune cell subpopulations (Figure 4D) (for gating strategy see Supplementary Figure S7B). No differences between treatment groups were observed regarding the amount of CD4^+^ or CD8^+^ cells, but the calculation of the CD4/CD8 T cell ratio in the liver showed a shift to a more dominant CD4 related T cell response in mice fed with WD-NTF and WD-Corn. To elucidate these differences further, these CD4^+^ T cells were characterized in more detail. A significant increase in the amount of CD4^+^/CD25^+^ cells was evident in WD-NTF fed animals.

**FIGURE 5:**
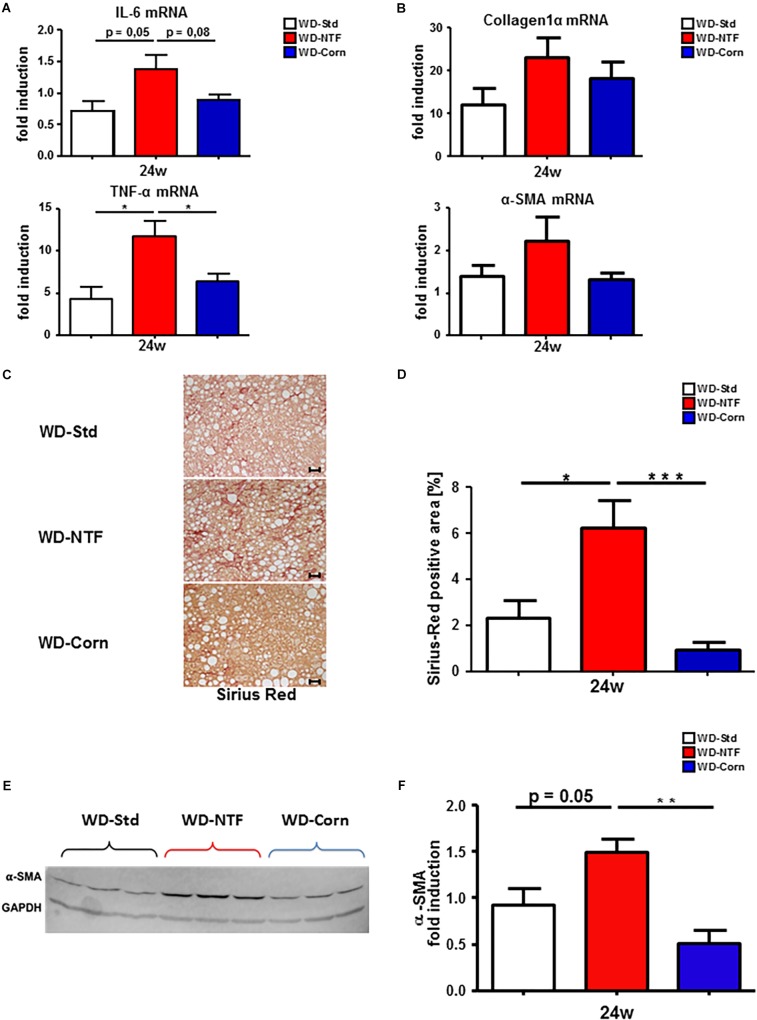
Earlier and stronger fibrosis development in WT animals fed with Western diet containing non-trans fat shortening as fat source. **(A)** mRNA expression levels of IL-6 and TNF-α. Whole liver homogenates of WT animals after 24 weeks treatment with Western diets containing different fat sources (WD-Std, WD-NTF, WD-Corn) were analyzed via real-time PCR. 4 animals per group were included (^∗^*p* < 0.05). **(B)** Analysis of mRNA expression levels of collagen1α and α-SMA in whole liver homogenates. WT mice were fed WD (WD-Std, WD-NTF, WD-Corn) for 24 weeks. mRNA expression was analyzed via real-time PCR. 4 animals per group were included. **(C)** Representative Sirius Red stained liver sections of WT animals fed with steatosis inducing Western diets (WD-Std, WD-NTF, WD-Corn). **(D)** Quantitative evaluation of Sirius Red stained liver sections of WD treated animals (*n* = 4) (^∗^*p* < 0.05, ^∗∗^ *p* < 0.01, ^∗∗∗^ *p* < 0.001). **(E)** α-SMA Western Blot analysis from whole liver extract of WT mice after treatment with WDs containing different fat sources (WD-Std, WD-NTF, WD-Corn). GAPDH serves as housekeeping protein. **(F)** Quantitative evaluation of α-SMA Western Blot analysis normalized to GAPDH expression levels. Analysis reveal a significant increase in protein levels of α-SMA in mice fed with WD containing non-trans fat shortening as fat source (*n* = 4) (^∗∗^*p* < 0.01).

The disparity of immune cell infiltration between the different treatments was studied further. Therefore, we performed immunofluorescent stainings for F4/80 and Ly6G/C to receive an impression of the distribution of F4/80^+^ cells (regarded as macrophages) and Ly6G/C^+^ cells (regarded as neutrophils). 24 weeks feeding with WD-NTF and WD-Corn led to an increase in F4/80^+^ cells (Figure 4E and Supplementary Figure S8A). This increase was clearly more pronounced in animals fed with WD-Corn compared to the other groups (WD-Std, WD-NTF). Conversely, Ly6G^+^ cells show a strong tendency to be increased in livers of animals receiving WD-NTF while mice fed WD-Corn display decreased levels compared to WD-Std and WD-NTF (Figure 4F and Supplementary Figure S8B). To specifically investigate whether these infiltrating cells contribute to a more pro-or anti-inflammatory microenvironment we performed mRNA expression analysis of different cytokines. Interestingly, experiments reveal an increased expression of the pro-inflammatory cytokines interleukin-6 (IL-6), TNF-α, MCP-1 and interleukin-18 (IL-18) in mice fed with WD-NTF (Figure 5A and Supplementary Figures S9A,B). Apart from that, WD-Std and WD-Corn fed animals exhibited similar results, although still elevated compared to normal chow fed WT animals, or even display a drop in cytokine expression (MCP-1, IL-18). This leads to the conclusion that WD-Std and WD-Corn display a comparable development and progression of diet-induced chronic liver injury whereas treatment with WD-NTF provokes a more pro-inflammatory milieu within the liver thereby resulting in more pronounced steatohepatitis. This observation can be corroborated when assessing the progression from simple fatty liver degeneration to fibrosis initiation by extracellular matrix accumulation as a result of hepatic stellate cell activation and transdifferentiation. All dietary treatments lead to elevated mRNA expression of collagen 1α and α-SMA, which is a hallmark of hepatic fibrogenesis and majorly driven by activated and transdifferentiated hepatic stellate cells (Figure 5B).

Animals after 24 weeks treatment with WD-NTF thereby show a trend to stronger expression of these genes compared to WD-Std and WD-Corn fed mice. Next, a Sirius Red staining was performed. This staining and the quantitative calculation of Sirius Red positive fibers demonstrate an escalation in fibrosis development in animals exposed to WD-NTF for 24 weeks (Figure 5C,D). Although the amount of the Sirius Red positive area also increases in mice fed WD for 24 weeks, these changes were more pronounced in WD-NTF treated animals. Surprisingly, WD-Corn did not lead to intrahepatic extracellular matrix deposition. These findings were further confirmed by the results of α-SMA Western Blot analysis which indicate stronger activation of hepatic stellate cells (Figure 5E,F).

### Treatment With Different Western Diets Is Associated With Diverse Inflammatory Conditions in eWAT

**FIGURE 6:**
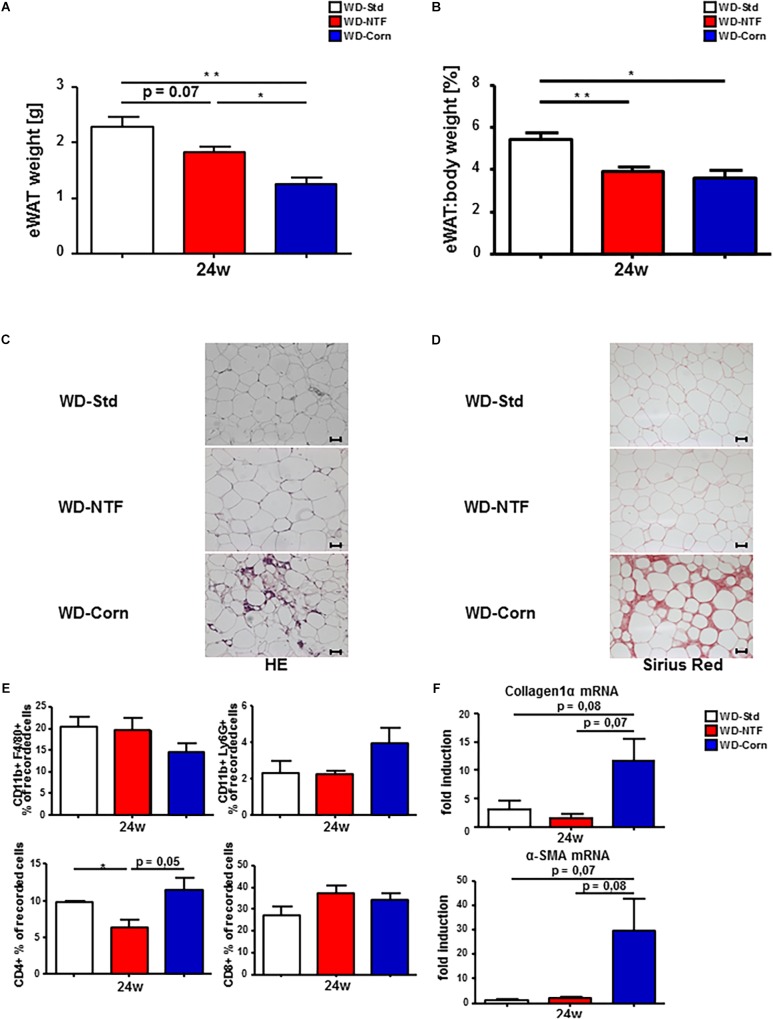
More pronounced fibrosis progression in eWAT of mice treated with WD containing corn oil. **(A)** Total eWAT weight and **(B)** eWAT:body weight ratio drops in WT animals fed a WD-NTF compared to standard WD-Std and WD-Corn after 24 weeks treatment (*n* = 4) (^∗^*p* < 0.05, ^∗∗^ *p* < 0.01). **(C)** Representative H&E stained eWAT sections of WT animals after 24 weeks treatment with different Western style diets (WD-Std, WD-NTF, WD-Corn). **(D)** Representative images of Sirius Red stained eWAT sections from WD fed WT animals (WD-Std, WD-NTF, WD-Corn). **(E)** Flow Cytometric analysis of eWAT infiltrating immune cells. Cells were gated via FSC/SSC, duplets were excluded. Live/CD45^+^, CD11b^+^/F4/80^+^ (regarded as macrophages) or CD11b^+^/Ly6G^+^ (regarded as neutrophils), CD4^+^ or CD8^+^ (*n* = 4) (^∗^*p* < 0.05). **(F)** mRNA expression levels of Collagen1α and α-SMA in whole eWAT homogenates. WT mice were fed WD (WD-Std, WD-NTF, WD-Corn) for 24 weeks. mRNA expression was analyzed via real-time PCR. 4 animals per group were included.

Western diets treatment leads to a significant increase in eWAT weight. The promoting influence of inflamed eWAT on NASH progression was described by Mulder et al. (2016). Thus, we next wanted to investigate whether the inflammatory differences observed in the liver upon treatment with different WDs is reflected in eWAT. Interestingly, total eWAT weight and the eWAT:body weight ratio were decreased in WD-NTF and WD-Corn compared to WD-Std (Figure 6A,B). In the course of this WD-Corn treated animals show the strongest drop in eWAT weight. Surprisingly, H&E and Sirius Red staining reveal that this is exactly the dietary treatment leading to immune cell infiltration and fibrosis development in animals after 24 weeks which does not become present in mice fed with WD-Std or WD-NTF (Figure 6C,D). Subsequent, flow cytometric analysis support these findings showing a trend to an increased cell infiltration of CD11b^+^/Ly6G^+^ and CD4^+^ cells in this group (Figure 6E). Consistent with the Sirius Red staining, mRNA expression of the fibrosis markers collagen 1α and α-SMA were overexpressed in mice after WD-Corn feeding (Figure 6F). These results suggest a great impact of corn oil on eWAT metabolism thereby maybe influencing steatosis and steatohepatitis development and progression in a positive way.

To get further insight into inflammatory changes, which may be associated with the diverse conditions in eWAT, the protein quantities of leptin and adiponectin were measured in serum, liver and eWAT directly. Interestingly, WT animals treated with different WDs (WD-Std, WD-NTF, WD-Corn) showed no differences in their leptin levels in serum and in the liver (Supplementary Figures S10A,B). At the same time eWAT leptin levels were significantly reduced in animals fed with WD-NTF (Supplementary Figure S10C). Adiponectin serum levels show a slight increase in animals fed with WT-Corn but do not change in the liver (Supplementary Figures S10D,E). eWAT on the contrary showed increased adiponectin levels in WT mice treated with WD-Corn (Supplementary Figure S10F).

## Discussion

Excessive intake of trans fats or industrially manufactured PUFA bears the risk to develop metabolic disorders such as obesity, which further lead to cardiovascular and hepatic diseases through promoting inflammation and oxidative stress (Monguchi et al., 2017). The mechanisms how the intake of industrially manufactured PUFA (trans fats) initiates metabolic disorders is well investigated. Recent studies describe a direct association between high serum levels of trans fatty acids and insulin resistance and the body weight composition in diabetic patients especially in the United States (Santos et al., 2013; Liu et al., 2018). In line with these results we found that feeding a trans fat containing WD to WT mice leads to an increase in body weight, an altered insulin resistance and other typical features of the metabolic syndrome finally leading to the development of NASH (Figure 1). Banning of theses trans fat supplementation to food products under which also the widely used Western diet (WD-Std) as animal model for steatohepatitis is affected lead us to critically reconsider this diet as appropriate model to mimic the human disease pathogenesis. Therefore, the aim of the present study was to evaluate the impact of different fat sources such as a NTF shortening and corn oil as supplementation to a high fat diet (HFD) to replace trans fats in the WD mouse model of steatohepatitis.

A variety of experiments in different rodent animal models showed that feeding a HFD containing different amounts of the most common PUFA n-3, n-6 and n-9 of natural origin modulate the inflammatory cytokine production (Pu et al., 2016; Sundaram et al., 2016). Cammisotto et al. (2003) could further find that long chain PUFA suppresses insulin-stimulated secretion of leptin which is crucial for the regulation of food intake and maintaining the metabolic homeostasis. This result was supported by a human study by St-Onge et al. (2014) which shows that long chain TG consumption instead of medium chain TG consumption does not reduce food intake by lower rise in leptin. These results are planned to be outlined in a multicentre interventional trial to investigate plasma fatty acid changes and cardiovascular disease risk factors in patients with abdominal obesity after consumption of different dietary oils (Senanayake et al., 2014). Recent studies controversially discuss the role of corn oil supplementation and even recommend replacing saturated fats by corn oil (Si et al., 2014).

Bohm et al. (2013) postulated in 2013 that food derived peroxidized fatty acids such as linoleic acid included in WD with corn oil increases hepatic lipid peroxidation and influences immune cells and other pro-inflammatory stimuli that trigger hepatic damage. These findings were corroborated by Takeuchi et al. (2000) and Slim et al. (1996); finding that corn oil supplementation increased hepatic cholesterol and TG levels and activated the oxidative stress response. On the contrary Si et al. (2014) showed 1 year later that a high corn oil diet improved health and longevity in mice by lowering serum lipid levels and decreasing the expression of pro-inflammatory cytokines such as IL-6, IL-1β or MCP-1. A recent study published by Ansar et al. (2017) supports this result by demonstrating that a substitution with corn oil and flaxseed oil reduces fat mass in HFD fed mice. In line with these results our study also shows a beneficial effect of dietary supplementation with corn oil in the WD mouse model compared to the former used WD-Std. WT mice treated with WD-Corn display less body weight gain, an improved insulin resistance and decreased serum lipid levels compared to “standard” trans fat WD (WD-Std) (Figure 1). Less pronounced steatohepatitis was further reflected by less histomorphological changes, less hepatic infiltration of neutrophils (CD11b^+^/Ly6G^+^) with simultaneous increase in the amount of monocytes (CD11b^+^/F4/80^+^) tending to secrete a decreased amount of pro-inflammatory cytokines (IL-6, TNF-α). This finally resulted in reduced fibrogenesis (Figure 4, 5). Surprisingly, WD-Corn treatment resulted in an increased immune cell infiltration and collagen accumulation in epididymal White Adipose Tissue (eWAT) (Figure 6). Others found in 2012 that lard in HFD also influences adipose tissue inflammation by regulating adipokine levels giving a direction for future investigations (Catta-Preta et al., 2012). While the more pro-inflammatory acting leptin was not changed in eWAT of mice fed WD-Corn in comparison to WD-Std, adiponectin, which is known to act in a more anti-inflammatory manner, was upregulated in eWAT of WD-Corn fed mice (Lopez-Jaramillo et al., 2014). During formation of metabolic syndrome this may act in a compensatory way, in which the leptin/adiponectin ratio and a potential imbalance of both mediators is highly critical.

On the other hand, treating mice with WD supplemented with a NTF shortening lead to a worsened disease progression and development compared to trans fat WD (WD-Std) in all parameters described before. Namely an increase in body weight, altered insulin resistance, enhanced serum lipid levels, severe histomorphological changes and increased steatohepatitis development (Figure 1–5). Further, a stronger pronounced progression toward fibrosis is depicted by an increased expression of inflammatory markers and collagen. Interestingly mice fed WD-NTF lack any observable changes in eWAT (Figure 6).

Whereat the effects of different fatty acid supplementations on steatosis development such as corn oil, olive oil, fish oil, walnut oil and many more were investigated in several different studies (Belzung et al., 1993; Couet et al., 1997; Flachs et al., 2006; Lozano et al., 2013; Sain et al., 2013) we could show for the first time, that the type of supplemented dietary fatty acids does not influence the lipid composition accumulating in liver tissue upon steatosis development (Figure 2, 3 and Supplementary Figure S4).

Taken together, our findings show that WD supplementation with corn oil shows comparable development and progression of steatohepatitis as WD-Std, whereas a WD supplemented with a NTF shortening leads to stronger steatohepatitis initiation and progression toward fibrosis. These findings fit perfectly to previous human studies showing that consuming of trans fats increase the risk of coronary artery disease by raising levels of low-density lipoprotein, lowering levels of high-density lipoproteins, and further increasing total amount of triglycerides, thereby promoting inflammation, oxidative stress and lipoperoxidation (Hadj Ahmed et al., 2018). Contrarily, typical corn oils contain only 0.25 g trans fat/100 g (Song et al., 2015) and are therefore less toxic and better for the overall metabolic situation.

Interestingly the type of fat source within the dietary treatment did not influence the lipid composition accumulating in the liver. The mixture of NASH-inducing diets available differs a lot and finding out the exact ingredients of each animal food with detailed information about the fatty acids composition is difficult. In this project the manufacturer has helped us greatly by providing us with the necessary detailed information (Supplementary Figure S4).

However, we must admit that our study has some limitations. In particular, the fact that the different WDs we used in our study still contain some compounds that are not classified in detail. For example, based on the distributor’s information, the different WDs (WD-Std, WD-NTF) contain Primex shortening. This is a mixture of moisture-free, refined and processed vegetable oils. The exact composition might change from batch to batch and is chemically not fully defined by each company. In addition, although power analysis justified the number of animals used in this study the number incorporated is small. This however, is in agreement with the actual guidelines of the new European animal welfare rules which are based on the 3R principle of Russell and Burch (Russell and Burch, 1959) of Replacement, Reduction and Refinement. This principle for more ethical use of animals in testing was implemented in the EU-Directive 2010/63 regarding the protection of animals used for scientific purposes in 2013 (Liedtke et al., 2013). Although in our study we only tested *n* = 4 in each group, our results show tendencies and statistically significant outcomes. This was only possible, because our study relied on highly standardized protocols in which every parameter (daily feed intake, weight, serum parameters, animal appearance etc.) was closely monitored. Therefore, we think that the current data from our study is highly relevant and can be generalized. Moreover, the study was urgently needed to allow to perform and compare future animal-based studies that need to be done without the usage of trans fats which were recently forbidden by the FDA in all human food products. Therefore, our study has an important translational character. In this regard, NAFLD studies using animals relying on WD are highly critical. There are numbers of comprehensive reviews highlighting this and other topics (Hansen et al., 2017; Denk et al., 2018; Jahn et al., 2018).

In sum, WD-NTF and WD-Corn are suitable to replace the treatment with “standard” WD-Std to investigate steatohepatitis and fibrosis development and progression in mice.

## Ethics Statement

This study was carried out in accordance with the recommendations of the Ethics of the regional authorities for nature, environmental and consumer protection of North Rhine-Westfalia (LANUV, Landesamt für Natur, Umwelt und Verbraucherschutz NRW) Recklinghausen, Germany, and approved by the LANUV Committee (Permit Number: TV11018G). The protocol was approved by the Ethics of the regional authorities for nature, environmental and consumer protection of North Rhine-Westfalia (LANUV, Landesamt für Natur, Umwelt und Verbraucherschutz NRW) Recklinghausen, Germany, and approved by the LANUV Committee (Permit Number: TV11018G).

## Author Contributions

HKD designed the study, acquired and analyzed the data, and drafted the manuscript. DCK designed the study, acquired funding, drafted the manuscript, and supervised the study. AB and AC acquired and analyzed the data. RW provided technical and material support, and wrote and revised the manuscript. AF, CH, EGSR, and PFH acquired and analyzed the data and critically revised the manuscript. RT revised the manuscript. CT critically revised the manuscript for intellectual content.

## Conflict of Interest Statement

The authors declare that the research was conducted in the absence of any commercial or financial relationships that could be construed as a potential conflict of interest.

## Footnotes

1 https://www.holmesreport.com/latest/article/trans-fat-issues-generate-online-buzz

2 http://www.lipidmaps.org/

3 http://mmcd.nmrfam.wisc.edu

## Supplementary Material

FIGURE S1 Composition WD-NTF. Composition of the used standard Western diet (WD-Std) (Primex Shortening as fat source) and the WD containing non-trans fat Primex Shortening as fat source.

FIGURE S2 Composition WD-Corn. Composition of standard Western diet (WD-Std) (Primex Shortening as fat source) compared to a WD containing corn oil as fat source.

FIGURE S3 Fatty acids composition of WDs. Comparison of the fatty acids composition of the different Western diets used.

FIGURE S4 Triglyceride composition. Comparison of TG by mass spectrometry imaging. Average spectra of animals receiving the WD-Std and WD-Corn show low variation in absolute peak intensity. The m/z range containing TG is highlighted.

FIGURE S5 Oil Red O staining and hepatic triglyceride levels. **(A)** Representative images of Oil Red O stained liver sections of WT animals after 24 weeks treatment with WD-Std, WD-NTF or WD-Corn. Scale bars 100 μm, magnification 200×. **(B)** Intrahepatic TG levels were determined in livers of WD-Std, WD-NTF or WD-Corn fed animals. 4 animals per group were included. (^∗^*p* < 0.05, ^∗∗^ *p* < 0.01, ^∗∗∗^ *p* < 0.001).

FIGURE S6 NAFLD activity score (NAS). Validation of the NAFLD activity score (NAS) considering steatosis grade, hepatocellular ballooning, lobular inflammation and oval cell hyperplasia. The histopathological validation was performed by Prof. Alain de Bruin at the University of Utrecht.

FIGURE S7 Immune cell gating strategy. **(A)** Representative gating of intrahepatic neutrophils and inflammatory macrophages of WT mice after WD treatment for 24 weeks. Cells were gated by FSC/SSC, duplets were excluded, live/dead/CD45^+^, CD11b^+^/Ly6G^+^ (neutrophils), CD11b^+^/F480^+^ (inflammatory macrophages). **(B)** Depicted is a representative gating of intrahepatic CD4^+^ and CD8^+^ cells in livers of WT animals fed with the different WDs over a 24 weeks time period. Cells were analyzed by gating on FSC/SSC, live/dead/CD45^+^, CD4^+^/CD8^+^. Duplets were excluded.

FIGURE S8 F4/80 and Ly6G staining. **(A)** Representative images of F4/80 stained liver sections of WT animals. Stainings were performed after 24 weeks of either chow or WD treatment (WD-Std, WD-NTF, WD-Corn). (Scale bars 100 μm, magnification 200×). **(B)** Representative Ly6G stained liver sections of WT mice after 24 weeks treatment with chow or WD (WD-Std, WD-NTF, WD-Corn) (Scale bars 100 μm, magnification 200×).

FIGURE S9 MultiPlex cytokine measurements. **(A)** MCP-1 or **(B)** interleukin-18 (IL-18) concentration in livers of WT mice fed with chow or different WDs for 24 weeks (WD-Std, WD-NTF, WD-Corn). Measurement of cytokine levels (*n* = 4) were performed with the Mouse serum MultiPlex Cytokine measurements kit Procarta 9-plex bead assay from Thermo Fisher Scientific (^∗^*p* < 0.05).

FIGURE S10 Leptin and Adiponectin levels in serum, liver and eWAT. **(A)** Serum, **(B)** intrahepatic and **(C)** eWAT leptin levels were measured in WT mice fed with different WDs for 24 weeks (WD-Std, WD-NTF, WD-Corn). Measurement of leptin levels (*n* = 4) were performed with the Mouse Leptin ELISA Kit from Sabbiotech (^∗^*p* < 0.05; ^∗∗^*p* < 0.01). **(D)** Serum, **(E)** intrahepatic and **(F)** eWAT adiponectin levels were measured in WT mice fed with different WDs for 24 weeks (WD-Std, WD-NTF, WD-Corn). Measurement of adiponectin levels (*n* = 4) were performed with the Mouse Adiponectin ELISA Kit from Rockland (^∗^*p* < 0.05; ^∗∗^ *p* < 0.01).

TABLE S1 Primary antibodies for immunofluorescent stainings.

TABLE S2 Oligonucleotides for real-time PCR analysis.

TABLE S3 Antibody panel used for FACS analysis.

TABLE S4 Protein lysis buffer for SDS PAGE.

## Abbreviations

α-SMA: α-smooth muscle actin
ALT: alanine aminotransferase
AST: aspartate aminotransferase
eWAT: epididymal white adipose tissue
FDA: food and drug administration
FFA: free fatty acid
HCC: hepatocellular carcinoma
HFD: high fat diet
IL-6: interleukin-6
KO: knockout
MCP-1: monocyte chemoattractant protein-1
NAFLD: non-alcoholic fatty liver disease
NASH: non-alcoholic steatohepatitis
NTF: non-trans fat
PUFA: polyunsaturated fatty acids
TG: triglyceride
TGF-β: transforming growth factor-β
TNF-α: tumor necrosis factor-α
WD: Western diet
WT: wildtype
