# Supplementary material for: The Influence of Different Fat Sources on Steatohepatitis and Fibrosis Development in the Western Diet Mouse Model of Non-alcoholic Steatohepatitis (NASH)
Source: Front Physiol. 2019 Jun 25;10:770. doi: 10.3389/fphys.2019.00770 (PMC6603084; doi:10.3389/fphys.2019.00770)

Supplementary Figure 1

**Product No.**	**D09100301**		**D16022301**	
	gm%	kcal%	gm%	kcal%
Protein	22	20	22	20
Carbohydrate	45	40	45	40
Fat	20	40	20	40
Total		100		100
kcal/gm	4,5		4,5	
**Igredient**	**gm**	**kcal**	**gm**	**kcal**
Casein, 80 Mesh	200	800	200	800
L-Cystine	3	12	3	12
Maltodextrin 10	100	400	100	400
Fructose	200	800	200	800
Sucrose	96	384	96	384
Cellulose, BW 200	50	0	50	0
Soybean Oil	25	225	25	225
**Primex Shortening**	**135**	1215	**0**	0
**Primex, 101650, non trans fat**	**0**	0	**135**	1215
**Lard**	20	180	20	180
Mineral Mix S10026	10	0	10	0
DiCalcium Phosphate	13	0	13	0
Calcium Carbonate	5,5	0	5,5	0
Potassium Citrate, 1 H2O	16,5	0	16,5	0
Vitamin Mix V10001	10	40	10	40
Choline Bitartrate	2	0	2	0
**Cholesterol**	**18**	0	**18**	0
FD&C Yellow Dye #5	0,05	0	0	0
FD&C Red Dye #40	0	0	0,05	0
FD&C Blue Dye #1	0	0	0	0
**Total**	**904,05**	**4056**	**904,05**	**4056**
Taken from Broogarden ®	**WD-Std**		**WD-NTF**	

Supplementary Figure 2

Product No.	D09100301		D16010101	
	gm%	kcal%	gm%	kcal%
Protein	22	20	22	20
Carbohydrate	45	40	45	40
Fat	20	40	20	40
Total		100		100
kcal/gm	4,5		4,5	
**Igredient**	**gm**	**kcal**	**gm**	**kcal**
Casein, 80 Mesh	200	800	200	800
L-Cystine	3	12	3	12
Maltodextrin 10	100	400	100	400
Fructose	200	800	200	800
Sucrose	96	384	96	384
Cellulose, BW 200	50	0	50	0
Soybean Oil	25	225	25	225
**Primex Shortening**	**135**	1215	**0**	0
**Corn Oil, Partially Hydrogenated**	**0**	0	**135**	1215
**Lard**	20	180	20	180
Mineral Mix S10026	10	0	10	0
DiCalcium Phosphate	13	0	13	0
Calcium Carbonate	5,5	0	5,5	0
Potassium Citrate, 1 H2O	16,5	0	16,5	0
Vitamin Mix V10001	10	40	10	40
Choline Bitartrate	2	0	2	0
**Cholesterol**	**18**	0	**18**	0
FD&C Yellow Dye #5	0,05	0	0,025	0
FD&C Red Dye #40	0	0	0	0
FD&C Blue Dye #1	0	0	0,025	0
**Total**	**904,05**	**4056**	**904,05**	**4056**
Taken from Broogarden ®	**WD-Std**		**WD-Corn**	

Supplementary Figure 3

Typical FA Compositions of D09100301, D16010101 and D16022301

	D09100301	D16010101	D16022301
Total	180	180	180
C2, Acetic	0.0	0.0	0.0
C4, Butyric	0.0	0.0	0.0
C6, Caproic	0.0	0.0	0.0
C8, Caprylic	0.0	0.0	0.0
C10, Capric	0.0	0.0	0.0
C12, Lauric	0.3	0.0	0.6
C14, Myristic	0.6	0.3	1.7
C14:1, Myristoleic, n-9	0.0	0.0	0.0
C15	0.0	0.0	0.0
C16, Palmitic	**29.8**	**13.6**	**63.4**
C16:1, Palmitoleic, n-9	0.3	0.3	0.3
C16:2, n-4	0.0	0.0	0.0
C16:3, n-9	0.0	0.0	0.0
C16:4, n-4	0.0	0.0	0.0
C17	0.2	0.1	0.1
C17:1	0.1	0.0	0.0
C18, Stearic	14.4	20.0	12.7
C18:1, Oleic, n-9, Cis	58.9	65.4	63.4
C18:1, Elaidic, Trans	34.0	45.8	0.5
C18:2, Linoleic	26.6	19.7	31.3
C18:2, Trans	5.4	1.8	0.3
C18:3, Linolenic	2.3	2.1	2.5
C18:3, n-6	0.0	0.0	0.0
C18:3, Trans	0.2	0.6	0.1
C18:4, Stearidonic	0.0	0.0	0.0
C20, Arachidic	0.6	1.2	0.7
C20:1	0.4	0.9	0.2
C20:2	0.2	0.2	0.2
C20:3, n-6	0.0	0.0	0.0
C20:3, n-3	0.0	0.0	0.0
C20:4, Arachidonic, n-6	0.1	0.1	0.1
C20:4, n-3	0.0	0.0	0.0
C20:5, Eicosapentaenoic, n-3	0.0	0.0	0.0
C21:5, n-3	0.0	0.0	0.0
C22, Behenic	0.5	0.5	0.1
C22:1, Erucic	0.0	0.0	0.0
C22:4, Clupanodonic, n-6	0.0	0.0	0.0
C22:5, Docosapentaenoic, n-3	0.0	0.0	0.0
C22:6, Docosahexaenoic, n-3	0.0	0.0	0.0
C24, Lignoceric	0.2	0.4	0.0
C24:1	0.0	0.0	0.0
Total Trans Fat	39.6	48.1	0.9
Total	175.2	172.8	178.1
Saturated (g)	46.7	36.0	79.3
Monounsaturated (g)	59.7	66.6	63.8
Polyunsaturated (g)	29.2	22.1	34.1
Saturated (%)	26.6	20.8	44.5
Monounsaturated (%)	34.1	38.5	35.8
Polyunsaturated (%)	16.7	12.8	19.1
Trans (%)	22.6	27.9	0.5

Supplementary Figure 4

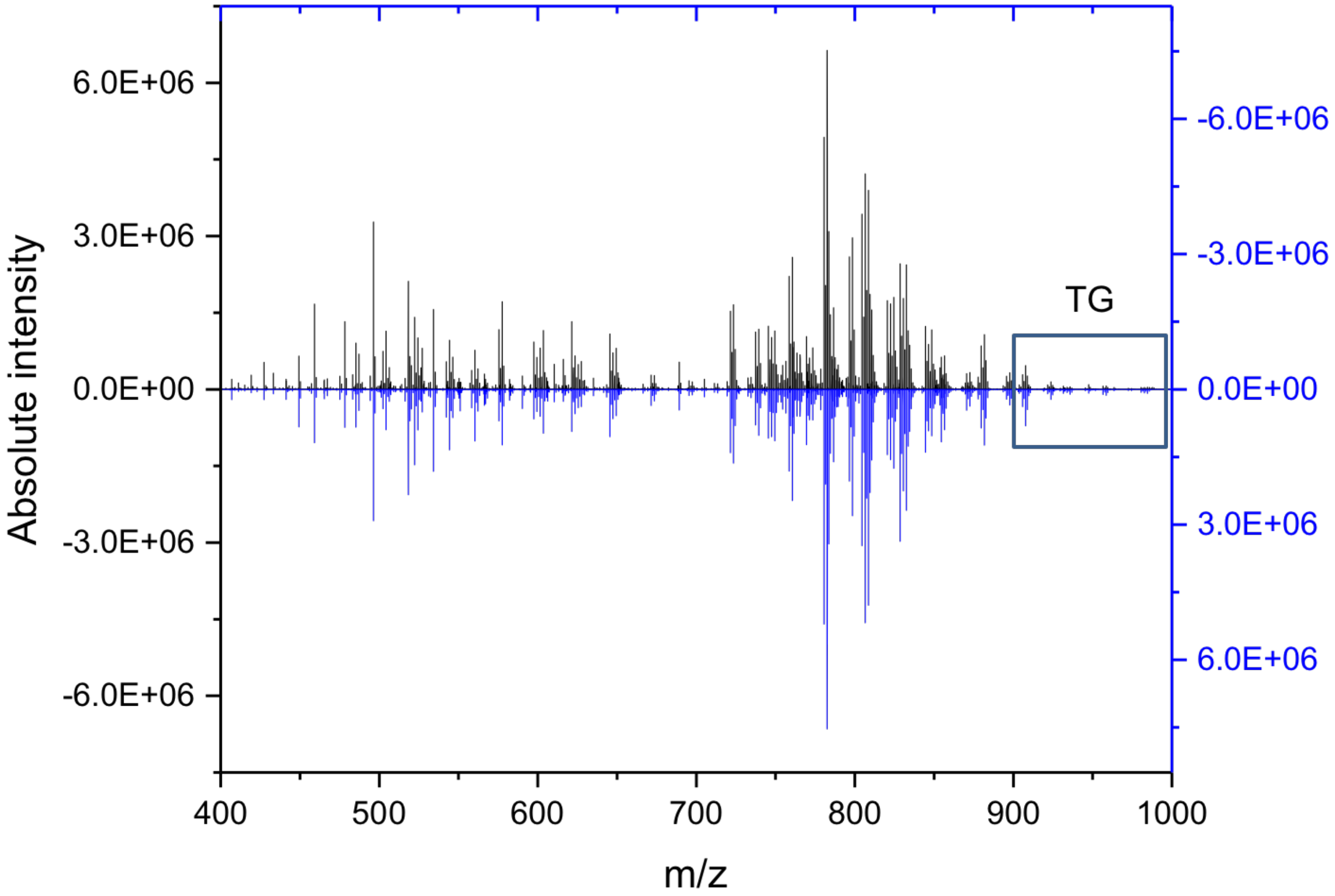

Supplementary Figure 5

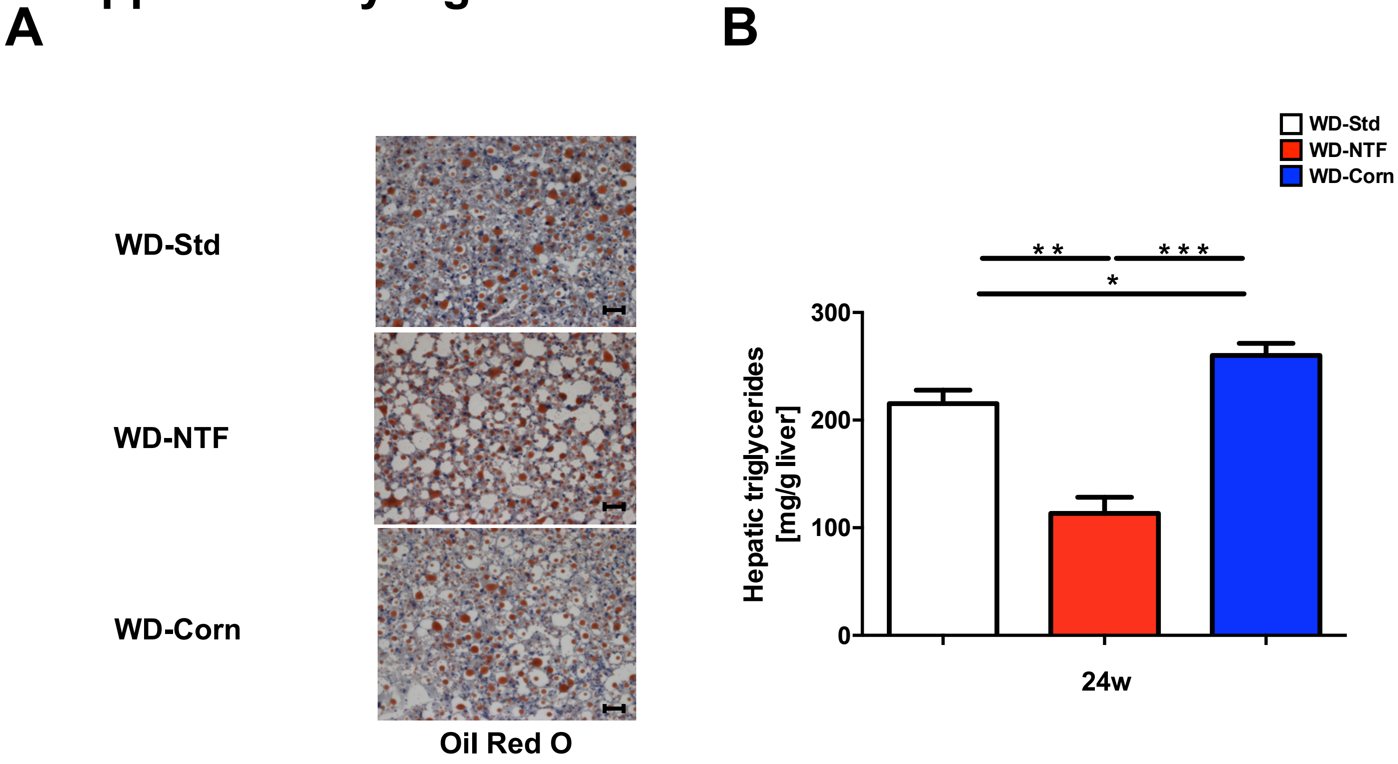

Supplementary Figure 6

DMPC 2017-1020
Representative pictures

	WD (cases B6/1-4)	WD non trans fat (cases B6/5-8)	WD corn oil (Cases B6/9-12)
	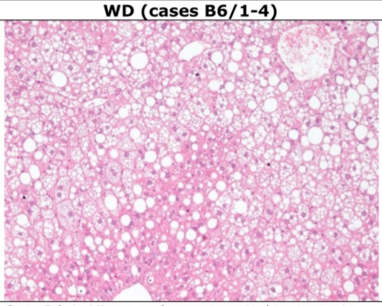	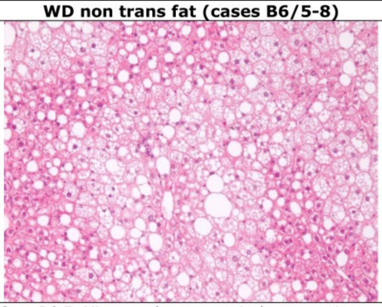	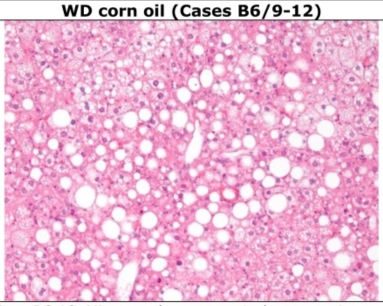
	Case B6-1. Micro- and macro-vesicular steatosis. 10X.	Case B6-7. Micro- and macro-vesicular steatosis. 10X.	Case B6-10. Micro- and macro-vesicular steatosis. 10X.
**Steatosis grade**	**3**	**3**	**3**
**MI%**	**58%**	**25%**	**31%**
	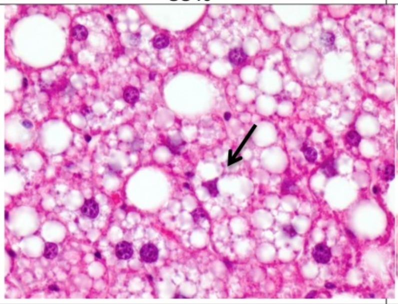	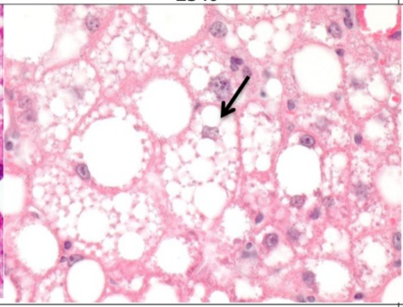	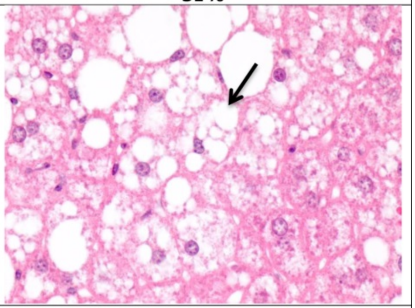
	Case B6-4. Ballooning hepatocyte (arrow). 40X.	Case B6-7. Ballooning hepatocyte (arrow). 40X.	Case B6-9. Ballooning hepatocyte (arrow). 40X.
**Ballooning**	**1**	**0,25**	**1**

DMPC 2017-1020
Representative pictures

	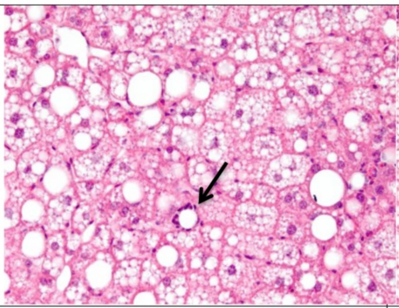	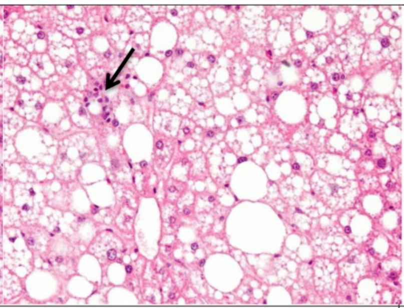	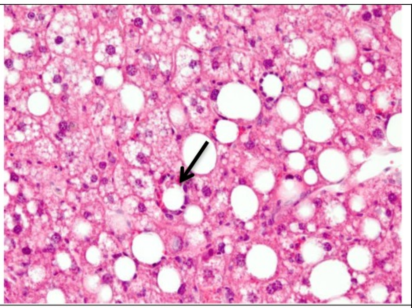
	Case B6-4. Lobular inflammation around macrovesicular steatotic hepatocyte (arrow). 20X.	Case B6-7. Lobular inflammation (arrow). 20X.	Case B6-12. Lobular inflammation around macrovesicular steatotic hepatocyte (arrow). 20X.
**Lobular Inflammation**	**1,75**	**1,25**	**1**
	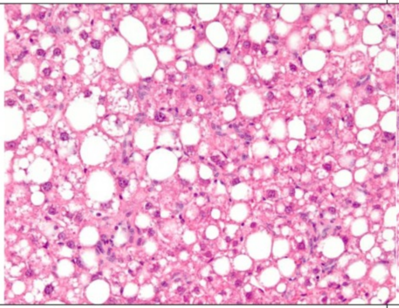	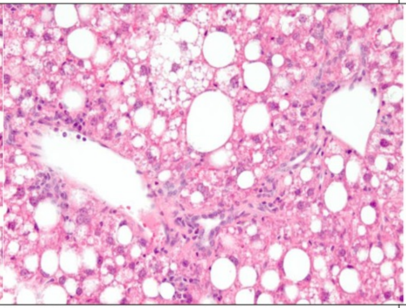	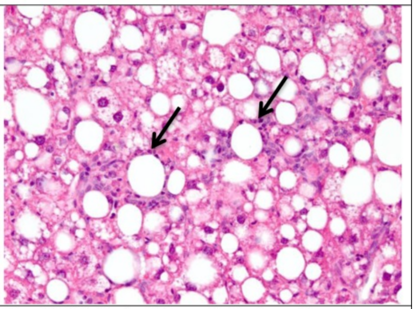
	Case B6-3. Multifocal foci of oval cell hyperplasia stage 1A. 20X.	Case B6-8. Multifocal foci of periportal oval cell proliferation. 20X	Case B6-10. Oval cell hyperplasia stage 1A and multifocal lobular inflammation (arrows). 20X.
**Oval cell hyperplasia**	**1**	**2**	**1,5**
**NAFLD**	**5,75**	**4,5**	**5**

Supplementary Figure 7

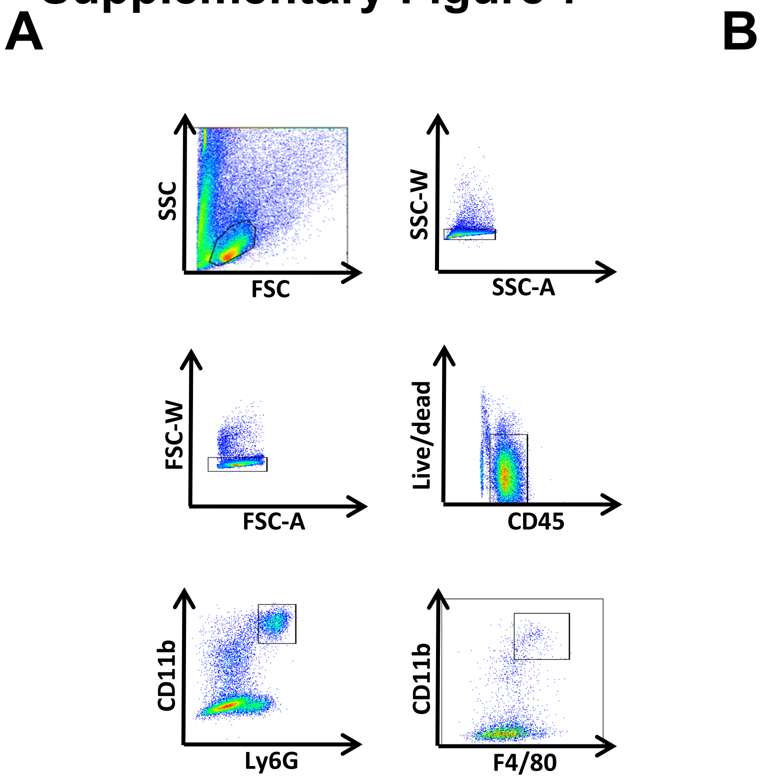

Supplementary Figure 8

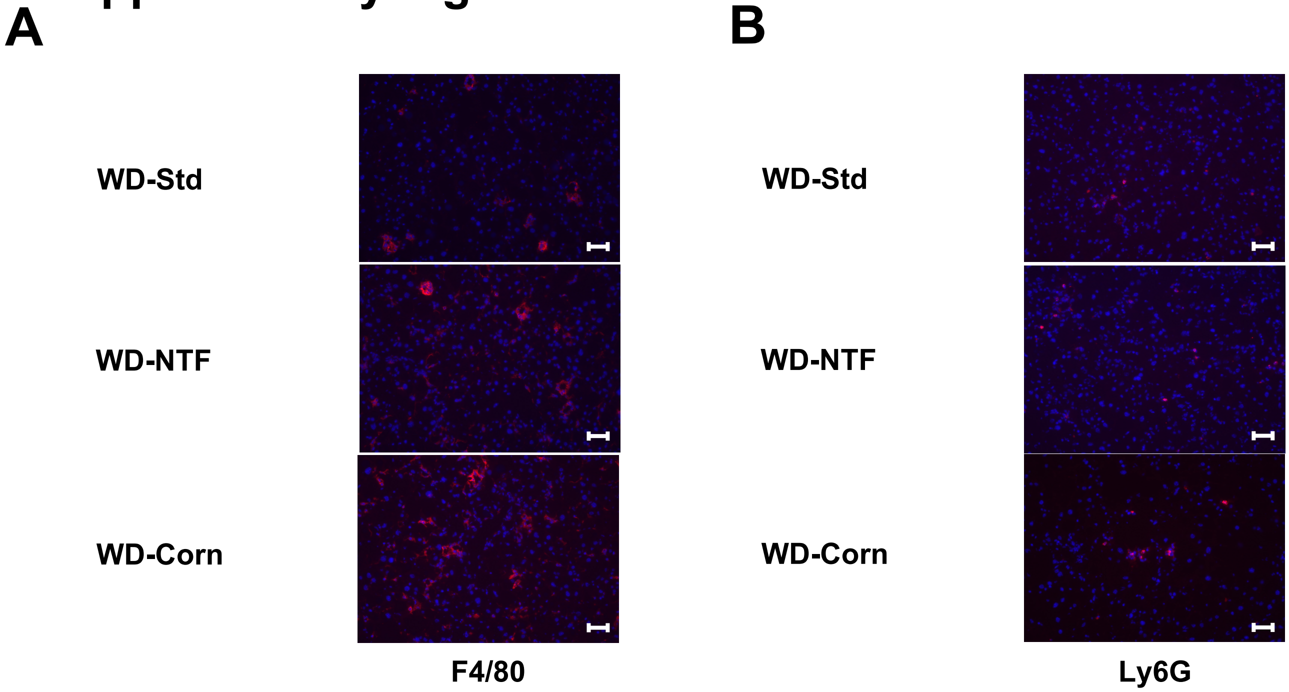

Supplementary Figure 9

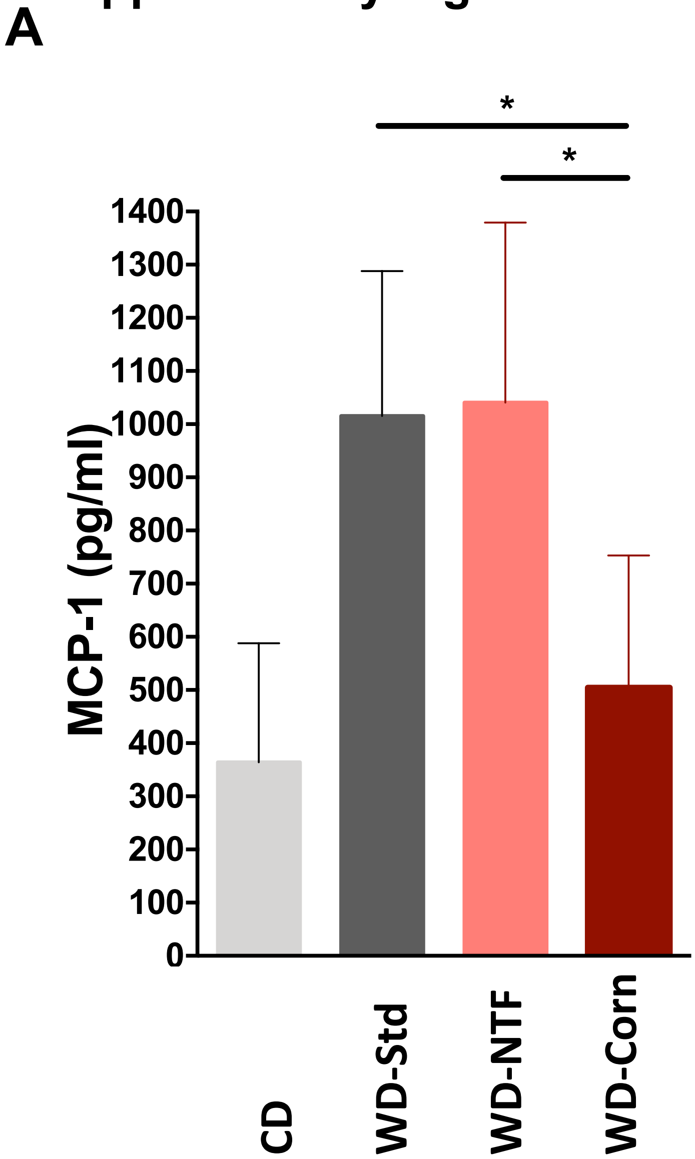

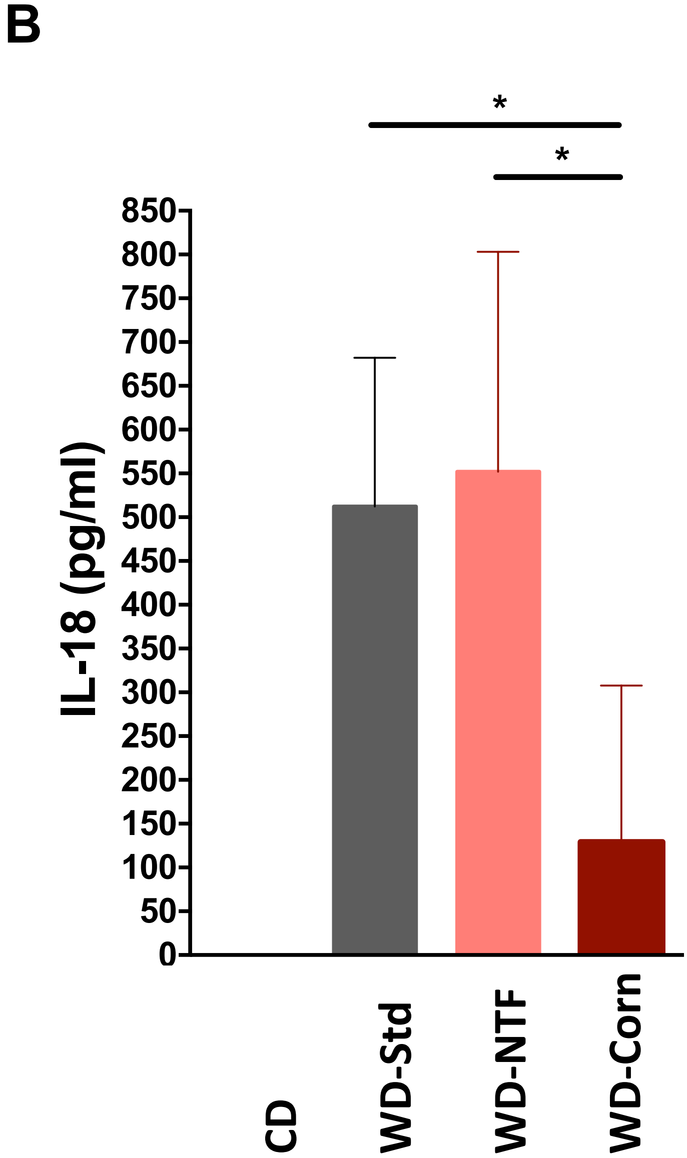

Supplementary Figure 10

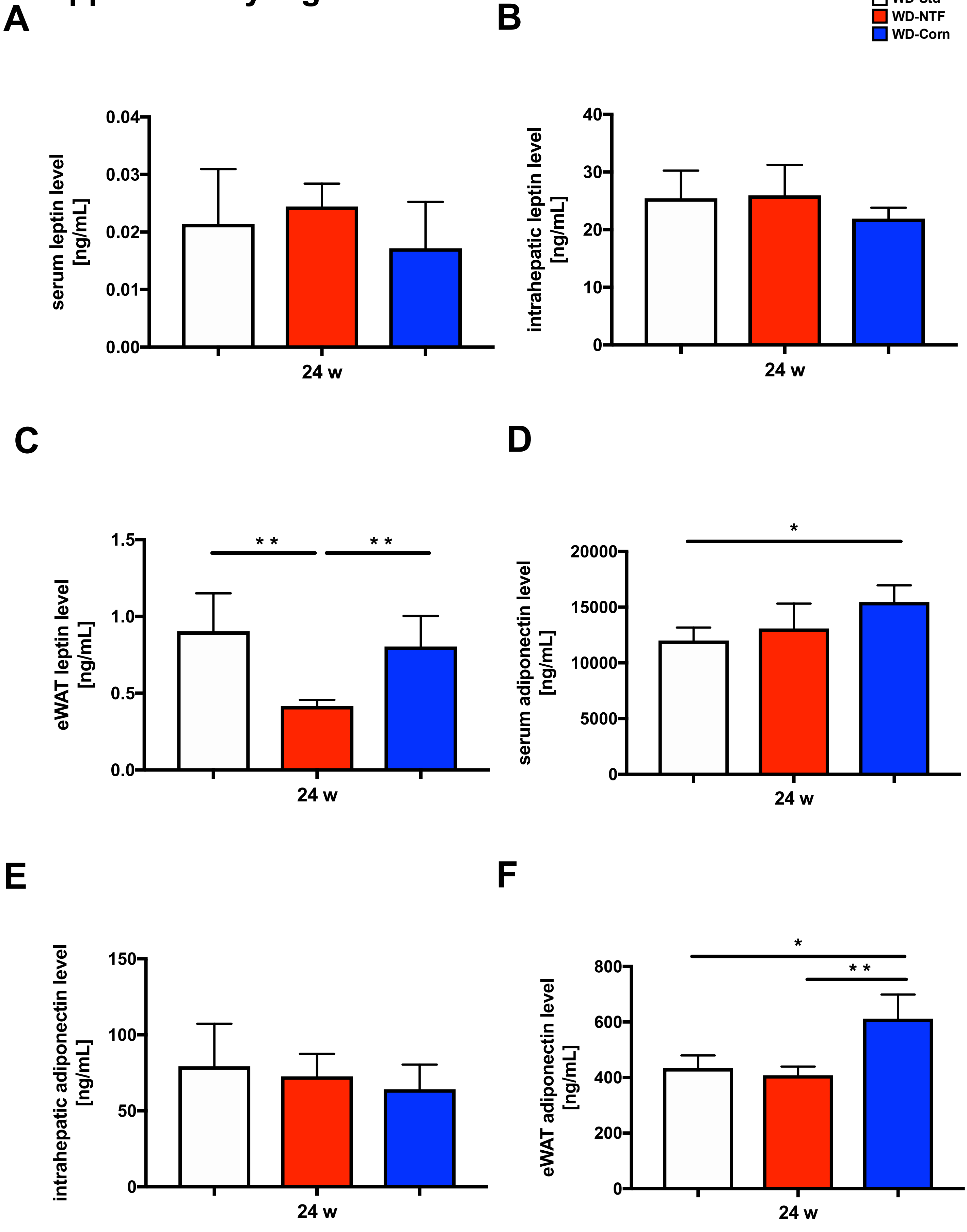

Supplement: FIGURE S1 — Composition WD-NTF. Composition of the used standard Western diet (WD-Std) (Primex Shortening as fat source) and the WD containing non-trans fat Primex Shortening as fat source. [file Data_Sheet_2.pdf]
