# Supplementary material for: The Influence of Different Fat Sources on Steatohepatitis and Fibrosis Development in the Western Diet Mouse Model of Non-alcoholic Steatohepatitis (NASH)
Source: Front Physiol. 2019 Jun 25;10:770. doi: 10.3389/fphys.2019.00770 (PMC6603084; doi:10.3389/fphys.2019.00770)
Supplement: TABLE S1 — Primary antibodies for immunofluorescent stainings. [file Data_Sheet_1.docx]

**The influence of different fat sources on steatohepatitis and fibrosis development in the Western diet mouse model of non-alcoholic steatohepatitis (NASH)**

**Table of contents:**

[Supplementary Material and Methods: 2](#_Toc8830979)

[Blood collection 2](#_Toc8830980)

[AST/ALT analysis 2](#_Toc8830981)

[NAFLD activity score 2](#_Toc8830982)

[Glucose tolerance test 2](#_Toc8830983)

[Hepatic triglycerides 2](#_Toc8830984)

[Leptin and Adiponectin ELISA 2](#_Toc8830985)

[Histology, Sirius-red staining, Oil Red O staining 3](#_Toc8830986)

[Immunofluorescent stainings 3](#_Toc8830987)

[Gene expression analysis by real-time PCR 4](#_Toc8830988)

[Mouse serum MultiPlex cytokine measurements 4](#_Toc8830989)

[Isolation of cells and flow cytometry 4](#_Toc8830990)

[SDS PAGE and Western blot 4](#_Toc8830991)

[Supplementary Tables: 6](#_Toc8830992)

[Supplementary Table 1: Primary antibodies for immunofluorescent stainings 6](#_Toc8830993)

[Supplementary Table 2: Oligonucleotides for real-time PCR analysis 7](#_Toc8830994)

[Supplementary Table 3: Antibody panel used for FACS analysis 8](#_Toc8830995)

[Supplementary Table 4: Protein lysis buffer for SDS PAGE 9](#_Toc8830996)

[References 10](#_Toc8830997)

# Supplementary Material and Methods:

## Blood collection

Blood collection was performed retro-orbital. Therefore, mice were shortly anaesthetised with isoflurane and blood was collected via a glass capillary. Samples were centrifuged at 10.000 rpm for 5 min and serum was stored at −80°C.

## AST/ALT analysis

Serum transaminase levels (aspartate aminotransferase (AST) and serum alanine aminotransferase (ALT)) were processed by the Central Laboratory Facility (LDZ) of the University Hospital RWTH Aachen.

## NAFLD activity score

Histopathological scoring was done by Prof. Dr. Alain de Bruin at the University of Utrecht via a NAFLD activity score (NAS) as described by Kleiner *et al.*[[15](#_ENREF_15)] and Hubscher *et al.*[[16](#_ENREF_16)].

## Glucose tolerance test

Animals were fasted for 6 hours and blood glucose was measured every 30 min following intraperitoneal administration of 0.2 g/kg glucose for 2 hours.

## Hepatic triglycerides

Hepatic triglyceride levels were analyzed by weighing 20 mg snap frozen liver tissue. This tissue was homogenized in 1 ml of buffer (10 mM Tris, 2 mM EDTA, 0.25 M sucrose, pH 7.5). A standard curve was calculated in accordance with the manufacturer’s instructions of the Instruchemie liquicolor mono Kit (Instruchemie, Delfzijl, Netherlands). 200 µl of the kit reagent were added to 2 µg of the sample or the standard solution and incubated for 45 min at room temperature. After that the OD was measured at 492 nm.

## Leptin and Adiponectin ELISA

Mouse leptin levels were measured in serum, liver and eWAT tissue. Serum was used in a 1:50 dilution. Liver and eWAT were lysed 100 mg/mL in ice cold PBS followed by centrifugation at 5000 x g for 5 minutes. The supernatant was measured in a 1:5000 dilution for liver tissue and 1:500 for eWAT tissue. ELISA measurement was performed in accordance to the manufacturer’s instruction (Mouse Leptin ELISA Kit, Cat No EK1237, Sabbiotech, College Park, MD, USA).

Mouse Adiponectin levels were measured in serum, liver and eWAT tissue. Serum was used in a 1:4000 dilution. Liver and eWAT were lysed 100 mg/mL in ice cold PBS followed by centrifugation at 5000 x g for 5 minutes. The supernatant was measured in a 1:100 dilution for liver and eWAT tissue. ELISA measurement was performed in accordance to the manufacturer’s instruction (Mouse Adiponectin ELISA Kit, Cat No KOA0366, Rockland Immunochemicals Inc, Pottstown, PA, USA)

## Histology, Sirius-red staining, Oil Red O staining

Liver samples were fixed in 4% formaldehyde, embedded in paraffin, cut and stained with hematoxylin and eosin. Pictures were taken using an Axio-Imager Z1 (Carl Zeiss, Jena, Germany) for each treatment per genotype.

For Sirius-red staining the paraffin sections were incubated in a solution containing 0.1% Sirius red and 0.1% picric acid (pH 2.0) for 1 hour. After that slides were incubated in 0.1 M HCl for 5 min, treated with an ascending ethanol series and finally incubated and covered in Roti-Histol (Roth, Karlsruhe, Germany). 10 images per liver section were analyzed under polarised light. Photomicrographs of Sirius-red positive areas taken in a 400x magnification were analyzed via colour error measurement using the open source software Image-J.

For Oil Red O staining formalin fixed frozen liver sections were washed in PBS and stained with Oil Red O staining solution (Sigma Aldrich, Cat. No. O1391). After rinsing with water counterstaining of nuclei was performed with haematoxylin.

## Immunofluorescent stainings

Liver tissue samples conserved in OCT compound were cut in 5-mm sections, air‑dried and fixed with ice-cold 4% paraformaldehyde. Antibodies (Supplementary Table 1: Primary antibodies for immunofluorescent stainings) were incubated in 1% mouse serum dissolved in PBS (PAA, Vienna, Austria) containing 0.02% sodium acetate (Sigma Aldrich) for 1 hour at room temperature. AlexaFluor 488-conjugated or Alexa Fluor 594-conjugated secondary antibodies (Molecular Probes, Boston, MA, USA) were used for detection. Nuclei were counterstained with DAPI (Vector Laboratories/Axxora, Loerrach, Germany). Immunofluorescent signals were detected using an AxioImager Z1 microscope (Carl Zeiss, Jena, Germany).

Images were taken in a 400x magnification. 10 photographs per mouse liver with at least 4 mice per group were counted.

## Gene expression analysis by real-time PCR

Total mRNA was extracted from whole cryopreserved liver tissue by using the peqGOLD RNAPure^TM^ kit (Peqlab, Erlangen, Germany) according to the manufacturer’s recommendations. For cDNA synthesis 500-1000 ng of total mRNA was first digested with DNAse I with the DNAse I kit (Invitrogen, Karlsruhe, Germany) and subsequently reverse-transcribed with the Omniscript reverse-transcription kit (Qiagen, Hilden, Germany) according to the manufacturer’s instructions and used in Real-Time PCR (Applied Biosystems, Foster City, CA). The detection of gene expression was performed by using the SybrGreen tqPCR Supermix (Invitrogen). Primer sequences can be found in Supplementary Table 2: Oligonucleotides for real-time PCR analysis. mRNA extracts from 4 mice per group were analyzed individually.

## Mouse serum MultiPlex cytokine measurements

Cytokines were measured in mouse sera using the chemokine 9-Plex mouse PorcartaPlex^TM^ bead assay (ThermoFisher Scientific, Schwerte, Germany) and a MAGPIX instrument using Luminex xMAP Technology. The analysis was essentially performed according to the manufacturer’s protocol.

## Isolation of cells and flow cytometry

Leukocytes were isolated from whole liver extracts. To analyze leukocytes in whole liver extracts livers were perfused with 10 ml phosphate-buffered saline (PBS), cut up with scissors and digested with collagenase IV (Worthington Biochemical Corporation, Lakewood, NJ, USA) at 37°C for 30 min. Digested liver extracts were filtered through a 70 µm cell strainer and cells were stained for 20 min at 4°C with fluorochrome-labeled monoclonal antibodies (used panel are depicted in Supplementary Table 3: Antibody panel used for FACS analysis).

Then, cells were subjected to flow cytometry using a BD Fortessa (BD Biosciences, Heidelberg, Germany). Data were analyzed using FlowJo software (TreeStar, Ashland, OR, USA).

## SDS PAGE and Western blot

Tissue of snap frozen livers was lysed in ice cold lysis buffer (Supplementary Table 4: Protein lysis buffer for SDS PAGE). The protein lysat was heat denaturated for 5 min. at 95°C in double-strength sodium dodecyl sulfate sample buffer containing dithiothreitol before resolution in 10% SDS-PAGE. For primary antibody incubation membranes were probed with anti-α-SMA (ab32575, Abcam, Cambridge, UK), and anti-GAPDH (MCA4739, AbD serotec, Hercules, CA, USA) antibodies.

As secondary antibodies HRP-linked anti-rabbit immunoglobulin G (7074S, Cell signaling, Frankfurt, Germany) and HRP‑linked anti-rat (559286, BD Pharmingen, Heidelberg, Germany) were used. The antigen-antibody complexes were visualized using the ECL chemiluminescence kit (GE Healthcare, Buckinghamshire, UK).

# Supplementary Tables:

## Supplementary Table 1: Primary antibodies for immunofluorescent stainings

| Immunoreactivity | Dilution | Species | Manufacturer / Order number |
| --- | --- | --- | --- |
| F4/80  Ly6G | 1:100  1:100 | rat  rat | BD Pharmingen, Heidelberg, Germany (Cat. No 557659)  BD Pharmingen (Cat. No 557659) |

## Supplementary Table 2: Oligonucleotides for real-time PCR analysis

| Gene | Oligonucleotide sequence |
| --- | --- |
| GAPDH | *forward:* TGT TGA AGT CAC AGG AGA CAA CCT  *reverse:*  AAC CTG CCA AGT ATG ATG ACA TCA |
| TGF-β | *forward:*  CAG CTT TGC TCA CTG CAG TAC A  *reverse:*  CTT GGC CGC CTC TAA CGA TA |
| TNF-α | *forward:* AGC ACA GAA AGC ATG ATC CG  *reverse:* CCC GAA GTT CAG TAG ACA GAA GAG |
| IL-6 | *forward:* CTG CAA GAG ACT TCC ATC CAG  *reverse:* AGT GGT ATA GAC AGG TCT GTT GG |
| MCP1 | *forward:* TCA GCC AGA TGC AGT TAA CG  *reverse:* AAC TAC AGC TTC TTT GGG ACA C |
| α-SMA | *forward:* ATG AAG CCC AGA GCA AGA GA  *reverse*: ATG TCG TCC AGT TGG TGA T |
| Collagen1α | *forward:* GCT ACT ACC GGG CCG ATG ATG C  *reverse:* CCT TCG GGG CTG CGG ATG TTC |

Note: All primers were purchased from MWG, Ebersberg, Germany.

## Supplementary Table 3: Antibody panel used for FACS analysis

| Panel | Antibody (anti-mouse) | Conjugate | Manufacturer / Order number |
| --- | --- | --- | --- |
| panel 1 | CD45 | APC-Cy7 | BD Pharmingen, Heidelberg, Germany  (Cat. No 557659) |
|  | F4/80 | APC | eBioscience, Santa Clara, CA, USA  (Cat. No 47-4801) |
|  | CD11b | PE | eBioscience  (Cat. No 12-0112-82) |
|  | Ly6G | FITC | eBioscienceCA  (Cat. No 17-5931-81) |
|  | 7-AAD dye |  | Sigma Aldrich, St Louis, MO, USA  (Cat. No SML1633) |
| panel 2 | CD45 | APC-Cy7 | BD Pharmingen  (Cat. No 557659) |
|  | CD4 | FITC | eBioscience  (Cat. No 11-0042-82) |
|  | CD8 | PerCP-Cy5.5 | BD Pharmingen  (Cat. No 551162) |
|  | 7-AAD dye |  | Sigma Aldrich  (Cat. No SML1633) |

## Supplementary Table 4: Protein lysis buffer for SDS PAGE

| 1 M | NaCl |
| --- | --- |
| 0.01 M | EGTA |
| 0.5 M | EDTA (pH 8) |
| 1 M | NaH_2_PO_4_ |
| 1 M | Tris (pH 7.5) |
| 1 M | NaF |
| 10x | Triton |
| 0.1 M in EtOH | PMSF |
| 1 mM | Na_3_VO_4_ |
| In H_2_O dest. | |

# References

[1] Buettner R, Scholmerich J, Bollheimer LC. High-fat diets: modeling the metabolic disorders of human obesity in rodents. Obesity (Silver Spring) 2007;15:798-808.

[2] Yaqoob P, Sherrington EJ, Jeffery NM, Sanderson P, Harvey DJ, Newsholme EA, et al. Comparison of the effects of a range of dietary lipids upon serum and tissue lipid composition in the rat. Int J Biochem Cell Biol 1995;27:297-310.

[3] Alexander J, Chang GQ, Dourmashkin JT, Leibowitz SF. Distinct phenotypes of obesity-prone AKR/J, DBA2J and C57BL/6J mice compared to control strains. Int J Obes (Lond) 2006;30:50-59.

[4] West DB, Boozer CN, Moody DL, Atkinson RL. Dietary obesity in nine inbred mouse strains. Am J Physiol 1992;262:R1025-1032.

[5] Surwit RS, Kuhn CM, Cochrane C, McCubbin JA, Feinglos MN. Diet-induced type II diabetes in C57BL/6J mice. Diabetes 1988;37:1163-1167.

[6] Ramadori P, Weiskirchen R, Trebicka J, Streetz K. Mouse models of metabolic liver injury. Lab Anim 2015;49:47-58.

[7] Drescher HK, Schippers A, Clahsen T, Sahin H, Noels H, Hornef M, et al. beta7-Integrin and MAdCAM-1 play opposing roles during the development of non-alcoholic steatohepatitis. J Hepatol 2017;66:1251-1264.

[8] Idrissova L, Malhi H, Werneburg NW, LeBrasseur NK, Bronk SF, Fingas C, et al. TRAIL receptor deletion in mice suppresses the inflammation of nutrient excess. J Hepatol 2015;62:1156-1163.

[9] Beraza N, Malato Y, Vander Borght S, Liedtke C, Wasmuth HE, Dreano M, et al. Pharmacological IKK2 inhibition blocks liver steatosis and initiation of non-alcoholic steatohepatitis. Gut 2008;57:655-663.

[10] <https://www.jax.org/jax-mice-and-services/strain-data-sheet-pages/body-weight-chart-000664>. Last accessed: December 1, 2018 TJLBwifCBJJmsCBJSNAa.

[11] Faul F, Erdfelder E, Lang AG, Buchner A. G*Power 3: a flexible statistical power analysis program for the social, behavioral, and biomedical sciences. Behav Res Methods 2007;39:175-191.

[12] Charan J, Kantharia ND. How to calculate sample size in animal studies? J Pharmacol Pharmacother 2013;4:303-306.

[13] Kilkenny C, Browne WJ, Cuthill IC, Emerson M, Altman DG. Improving bioscience research reporting: The ARRIVE guidelines for reporting animal research. J Pharmacol Pharmacother 2010;1:94-99.

[14] Liedtke C, Luedde T, Sauerbruch T, Scholten D, Streetz K, Tacke F, et al. Experimental liver fibrosis research: update on animal models, legal issues and translational aspects. Fibrogenesis Tissue Repair 2013;6:19.

[15] Kleiner DE, Brunt EM, Van Natta M, Behling C, Contos MJ, Cummings OW, et al. Design and validation of a histological scoring system for nonalcoholic fatty liver disease. Hepatology 2005;41:1313-1321.

[16] Hubscher SG. Histological assessment of non-alcoholic fatty liver disease. Histopathology 2006;49:450-465.
